# Exercise Modalities for Improving Frontal Plane Knee and Foot Posture in Healthy Adults: A Systematic Review

**DOI:** 10.3390/sports13020052

**Published:** 2025-02-11

**Authors:** Gülsüm Mandir Cömert, Markus Gruber

**Affiliations:** Human Performance Research Centre, Department of Sport Science, University of Konstanz, 78464 Konstanz, Germany; m.gruber@uni-konstanz.de

**Keywords:** exercises, knee valgus, foot pronation, healthy individuals

## Abstract

Lower extremity misalignments increase the risk of chronic overload and acute injuries during sports and daily activities. Medial positioning of the knee and foot in the frontal plane is one of the key biomechanical risk factors associated with lower extremity injuries and pain. Different exercise interventions have been implemented to counteract misalignments. However, most studies have been conducted on clinical populations. Therefore, in this review, we aimed to assess the preventive effects of exercise interventions on frontal plane knee and foot posture in healthy individuals. Electronic databases (PubMed, Web of Science, PEDro) were systematically searched for original articles published between 2008 and 2024. This review included clinical trials on healthy adults (18–45 years) with or without lower extremity biomechanical misalignments, examining the effects of exercise interventions alone on knee and foot frontal plane biomechanics. Eligible studies reported at least one relevant frontal plane foot and knee biomechanical measure, such as knee valgus/abduction, medial knee displacement, foot pronation/eversion, or navicular drop. Studies involving non-exercise interventions, single-session protocols, and participants with neurological or spinal disorders, pain, or injury were excluded. A total of 35 articles with 1095 participants were included in this review. A total of 20 studies included individuals without a biomechanical misalignment, and 15 studies focused on individuals with a biomechanical misalignment. Mean values, standard deviations, and *p*-values were extracted from the included studies. Effect sizes and confidence intervals were then calculated to provide a quantitative presentation of the data. In conclusion, in healthy individuals without biomechanical misalignment, technique training and core muscles strengthening were most effective for improving knee valgus. Hip, core, and foot muscle strengthening reduced foot pronation in those with pronated feet, while short foot exercises improved foot positioning in individuals with flat feet. Combining lower extremity strengthening with knee position control training may reduce knee valgus in individuals with increased knee valgus.

## 1. Introduction

Poor posture and misalignments of the lower extremities pose a considerable risk of pain and injury during daily life activities for healthy individuals [1]. Medial positioning of the knee and foot in the frontal plane are the most prominent postural risk factors for lower extremities injuries and musculoskeletal pains [1,2,3]. In this regard, the terms abduction, valgus, eversion, and pronation have been used to describe medial movement in the frontal plane for the knee and foot joints [4]. Although these have been used to describe the knee and foot being positioned medially in the frontal plane, they essentially represent a comprehensive and complex definition with various components. When the knee joint moves away from the body’s midline, it refers to knee abduction. Thus, the knee turns inward in the frontal plane. Knee valgus is a multi-plane movement of the lower extremity, and it is a combination of adduction and internal rotation of the femur, abduction of the knee, anterior tibial translation, external tibial rotation, and ankle eversion [5]. Although some researchers use knee valgus and knee abduction interchangeably as similar terms, others agree that knee valgus is more complex than knee abduction [6]. Both lead to similar movement patterns and can cause similar problems [7]. The pelvis, hip, and femur are proximal contributors to knee positioning in the biomechanical chain. Increased anterior tilt of the pelvis, increased internal rotation, and adduction of the hip were related to increased knee valgus. In the distal segment, foot pronation increases the internal rotation of the tibia, and this causes knee valgus [8,9]. Similarly, in the opposite direction, knee valgus affects the movement of the calcaneus through the tibia and causes foot pronation [10]. Evidence suggests that excessive knee valgus increases the risk of medial knee stress, anterior cruciate ligament injuries, patellofemoral pain syndrome, knee osteoarthritis, and chronic injuries [11]. Foot posture is also crucial because it influences the alignment of the lower extremities through its joint connection with tibial rotation [12,13]. Foot pronation and knee valgus are both multiplanar movement complexes consisting of rearfoot eversion, forefoot abduction, and ankle dorsiflexion. It is defined as the foot rolling inward and the arch flattening. In some studies, foot eversion (i.e., ankle, calcaneal, or subtalar eversion) has also been used instead of foot pronation [14,15,16]. It refers to the outward tilting of the sole, moving the medial border of the foot downward and laterally. Rearfoot eversion, which is strongly related to tibial internal rotation, may be connected to hip transverse plane rotations [17,18]. With foot pronation in the early stance phase of the gait, when calcaneal eversion and talar adduction result in tibial and femoral internal rotation, this causes an increase in anterior pelvic tilt due to the tight fibrous connection at the sacroiliac joint [19]. This stress in the lumbopelvic region leads to low back pain [20,21]. There has long been a hypothesized connection between increased foot pronation and lower extremity injuries, based on this lower extremity joint linkage [22,23]. Coupling motions at the foot enhance internal tibial rotation at the knee, which can increase the risk of medial tibial stress syndrome [24] and knee osteoarthritis [25]. Researchers have reported that there is a connection between increased foot pronation and increased rotational and anterior knee laxity, and this is a risk for ACL injury [26,27]. Overpronation can also cause pain in the heel, arch, and Achilles, in addition to some deformities like hallux valgus or pes planus on the foot [28].

Exercise interventions have been widely used to improve lower extremity biomechanics and posture. Considering the fact that knee and foot biomechanics are affected by many factors, training programs vary in the literature. The effects of lower extremity strength exercises, as well as stabilization, plyometric, neuromuscular, and functional training on knee valgus/abduction and foot pronation/eversion, were intensively investigated in individuals with patellofemoral pain syndrome [29,30,31,32], ankle instability [33,34,35], and after ACL reconstruction [36,37,38]. Previous reviews also primarily focused on individuals with a specific pathological condition or a single biomechanical misalignment [39,40,41,42,43,44,45]. However, individuals with injuries or pain may have specific training needs and treatment programs to address their functional limitations. Thus, the results that were found in the studies mentioned above in a therapeutic setting cannot be directly transferred to healthy people and their needs in a preventive setting.

This systematic review aims to present an overview of studies on how exercise interventions could affect knee and foot medial positioning in healthy adults. The objective is to evaluate existing exercise programs, their goals, and results that can be integrated into daily life or sports programs to enhance lower extremity alignment and prevent injuries and chronic overload conditions in healthy individuals.

This systematic review focused on studies published between 2008 and 2024 to ensure the inclusion of the most relevant and current research in the field. Studies published after 2008 were analyzed due to the increased number and variety of exercise interventions and biomechanical measurement methods introduced in this period. The present review used knee valgus and foot pronation as representatives of all other terms used for similarly defined biomechanical alignments to identify medial positioning in the lower extremities.

## 2. Materials and Methods

### 2.1. Database Search

#### 2.1.1. Design

This systematic review followed the PRISMA (Preferred Reporting Items for Systematic Reviews and Meta-Analyses) guidelines and the Cochrane Handbook for Systematic Reviews of Interventions indications [46,47]. The PICO (**P**opulation, **I**ntervention, **C**omparison, **O**utcome) model was used to define our research question (Table A1) [48]. Healthy individuals aged 18–45 years old were the target population in the collected studies; exercises/training programs were the intervention; comparison was no training, sham training, or different types of training; and the outcomes were knee and foot frontal plane biomechanics.

#### 2.1.2. Eligibility Criteria

This review included clinical trials involving healthy adults aged 18 to 45 years with or without lower extremity biomechanical misalignments (valgus knee, pronated foot, flat foot, etc.). The studies needed to involve exercise interventions that were not combined with other conservative or invasive methods. Additionally, eligible studies were required to report at least one of the following outcome parameters to measure frontal plane knee and foot biomechanics: 3D or 2D knee valgus or knee abduction angles, knee frontal plane projection angle, medial knee displacement 3D or 2D foot pronation and eversion (abduction) angles, navicular drop, arch height, or foot posture index. Only the studies written in English and published as journal articles were eligible for the present review.

Studies involving children, elderly individuals, or participants with neurological or spinal disorders, extreme postural disorders (e.g., scoliosis), pain, or injury were not eligible for this review. Studies implementing non-exercise interventions, having unreported or missing data on relevant outcome parameters, or including exercise interventions limited to a single session or one-day protocols were excluded from this review. Studies written in languages other than English were also excluded.

#### 2.1.3. Search Strategy

In the present systematic review, electronic databases PubMed, Web of Science, and PEDro were used to obtain related original journal articles published between 2008 and 2024. The databases PubMed, Web of Science, and PEDro were chosen due to their comprehensive coverage of rehabilitation and exercise-related research. Additionally, the manual search involved screening the reference lists of included studies as well as review articles to ensure no relevant studies were omitted from the analysis. Non-indexed studies were excluded.

The search strategy was performed by two independent reviewers using Boolean operators (Table A2). Disagreements between the reviewers during the selection of the studies were resolved by consensus or by a third independent reviewer.

### 2.2. Study Selection and Data Extraction

**Figure 1:**
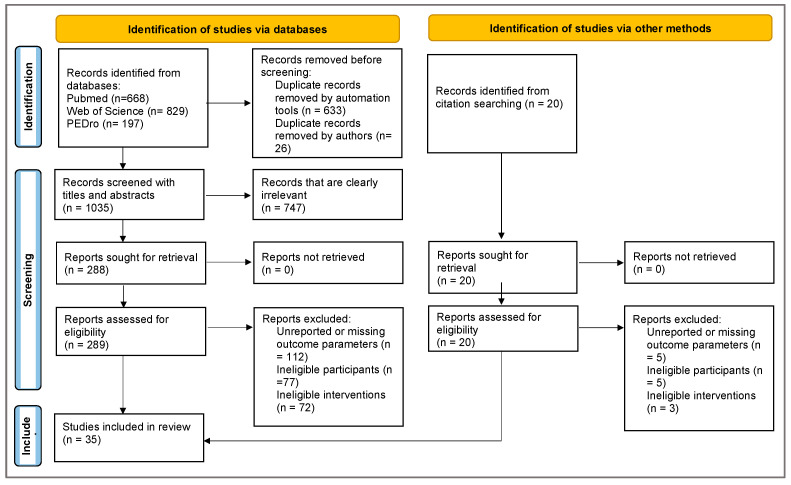
PRISMA flow diagram.

Potential studies were selected based on title and abstract initially. The Endnote reference manager tool [49] was used to eliminate duplicate results from different databases. After the first evaluation, two reviewers uploaded the articles selected according to the title and abstract to the Sciwheel online research management tool [50]. This tool allowed for the automatic elimination of duplicated studies. Each article was analyzed in full and categorized using tags, and the studies included in the systematic review were determined. The PRISMA [46] flow diagram was used to present information about the data collection and the steps of the included studies for this review (Figure 1).

The data extracted from each included study were synthesized detailing the first authors’ surnames, publication dates, sample sizes, participants’ ages and activity level, exercise interventions and their durations, group allocations, measurement methods, measurement tasks, and brief outcomes. Means, standard deviations (SD), and *p* values were extracted from the included studies to summarize the reported outcomes. The extracted data were categorized based on the presence or absence of biomechanical misalignment and presented in two separate tables (Supplementary Tables S1 and S2). Interventions were classified based on training modalities for each table.

### 2.3. Statistical Analysis

Effect sizes and confidence intervals were calculated from the included studies to gain a more precise understanding of the importance and reliability of the observed changes. Post hoc analyses were conducted to determine within-group and between-group effect sizes using G*Power (Version 3.1.9.3, University of Düsseldorf, Düsseldorf, Germany). For all studies, within-group effect sizes were estimated using a paired sample *t*-test. Between-group effect sizes were determined using an independent samples *t*-test for the studies with two groups and repeated measures ANOVA (between factors) for the studies with more than two groups. Cohen’s d was used for within-group and two-group comparisons, whereas Cohen’s f was used for multiple-group comparisons. Confidence intervals were calculated in Excel using the standard error of the effect size. The lower and upper limits were determined by subtracting and adding the product of the t-distribution critical value and the standard error to the effect size.

### 2.4. Risk of Bias Assessment

A Risk of Bias (RoB) assessment was conducted for the selected articles. RoB 2 Excel tool [51] was used for this assessment. The evaluation was made for all types of bias under 5 domain names including a randomization process, deviation from the intended interventions, missing outcome data, measurement of the outcome, and selection of the reported results. Individual and overall risk levels were reported.

## 3. Results

### 3.1. Study Selection

The electronic database search initially included 1694 potential studies. After removing duplicates using automation tools (633 duplicates) and manual verification by the authors (26 duplicates), 1035 records remained for screening. Titles and abstracts were screened, resulting in the exclusion of 747 clearly irrelevant records. This left 288 reports for full-text retrieval, all of which were successfully accessed. Out of the 289 studies assessed for eligibility, 112 studies were excluded due to unreported or missing outcome parameters, 77 studies due to ineligible participants, and 72 studies due to ineligible interventions. Additionally, citation searching identified 20 more records. From these, five were excluded due to unreported or missing outcome parameters, five due to ineligible participants, and three due to ineligible interventions. Finally, 35 studies with 1095 participants were included in this systematic review (Figure 1).

### 3.2. Data Extraction

The studies included in the review were summarized with their authors, objectives, participant profiles, interventions, measurement methods, and short results (Table 1).

In total, 22 of the studies included at least one control group and one intervention group. Six studies had only one intervention group, and the remaining six studies compared two intervention groups. In 15 studies, the groups consisted entirely of female participants, while in 3 studies the participants were exclusively male. In 17 studies, male and female participants formed the groups together. In 13 studies, participants had at least one lower extremity biomechanical misalignment (increased foot pronation, knee valgus, flat feet, limited ankle range of motion (RoM)). In 18 studies, the participants were athletes or recreationally active individuals, while in 17 studies the level of sport was not specified, or they were not physically active. In 19 studies, lower extremity biomechanics were measured with motion capture systems. Seven studies used digital cameras. The Foot Posture Index was used for measurements in six studies, and in eight studies, measurements were made with the navicular test. In total, 21 studies examined the effects of exercises on knee posture, and 12 studies focused on foot posture. In two studies, both knee and foot postures were measured.

The pre and post mean values, standard deviations, and *p*-values were extracted and presented based on participants’ biomechanical characteristics and training categories (Supplementary Tables S1 and S2).

**Figure 2:**
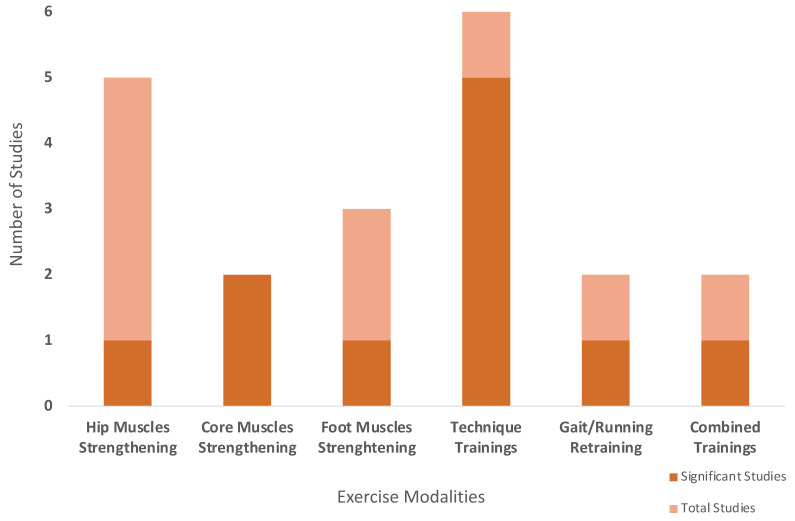
Significance of exercise modalities in individuals without a biomechanical misalignment in lower extremities.

In total, 20 studies were conducted on individuals without a lower extremity biomechanical misalignment (Figure 2). Hip muscle strengthening training was examined in seven studies, with five studies assessing knee valgus or knee abduction and two studies evaluating foot eversion. Only one study found a significant improvement in foot eversion. The effect of core muscle strengthening on knee valgus was investigated in two studies, and they both found a significant reduction following the intervention. Three studies focused on foot muscle strengthening and its effects on foot posture. Only one study reported a significant reduction in navicular drop. Technique training was implemented in six studies, evaluating its effects on knee valgus, knee abduction, and frontal plane projection angle (FPPA). Five out of six studies demonstrated significant improvements in knee alignment. In two studies examining the effects of gait/running retraining on foot pronation and eversion, only one study reported a significant reduction in foot pronation. Lastly, combined training programs were implemented in two studies, both evaluating knee abduction with one study reporting a significant decrease in knee abduction following the intervention (Figure 2).

**Figure 3:**
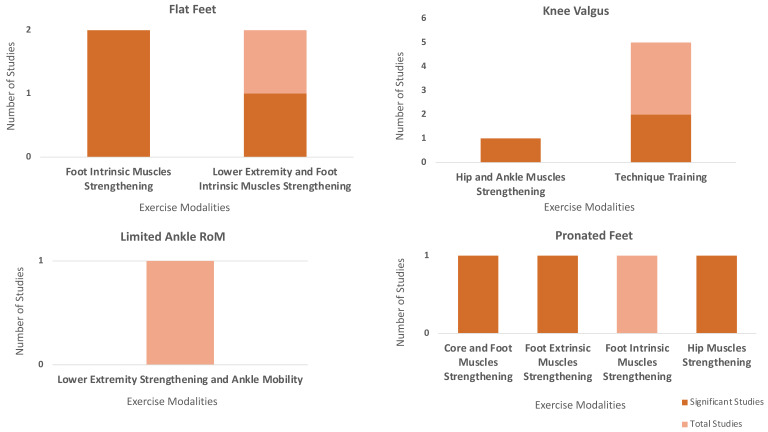
Significance of exercise modalities in individuals with a biomechanical misalignment in lower extremities.

A total of 15 studies examined different training interventions in individuals with knee valgus, limited ankle range of motion (ROM), pronated feet, and flat feet (Figure 3). Among the six studies focusing on knee valgus, five studies implemented technique trainings to evaluate the effect of the training on knee valgus, knee abduction, or the frontal plane projection angle (FPPA). Of these, two studies reported significant improvements in knee valgus and FPPA following training. Additionally, one study investigated the effects of hip and ankle muscle strengthening on knee posture in individuals with valgus knee and found significant reduction in knee valgus and medial knee displacement (MKD).

One study examined the effects of lower extremity strengthening and ankle mobility training on FPPA in individuals with limited ankle ROM but found no significant improvements.

There were four studies exploring the effects of different training programs in individuals with pronated feet. One study found that hip muscle strengthening significantly reduced navicular drop. One study reported a significant reduction in navicular drop following extrinsic foot muscle strengthening. Additionally, one study demonstrated that core and foot muscle strengthening led to a significant decrease in foot pronation. However, one study investigating intrinsic foot muscle strengthening found no significant changes in navicular drop or foot pronation.

Four studies evaluated the impacts of various training programs in individuals with flat feet. Two studies implemented intrinsic foot muscle strengthening trainings and found a significant reduction in foot pronation. There were two studies implemented extrinsic foot and lower extremity muscle strengthening, and only one study observed a significant reduction in navicular drop (Figure 3).

**Figure 4:**
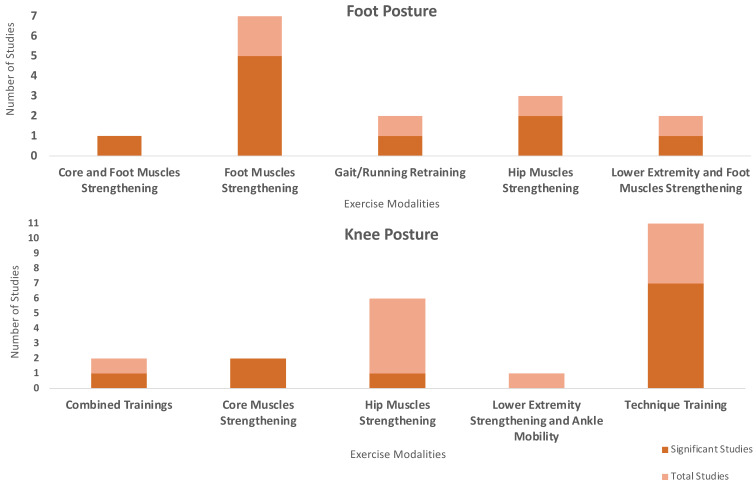
Significance of exercise modalities on foot posture and knee posture.

Finally, the number of studies that found statistically significant improvements in frontal plane knee and foot posture from the included studies is presented in Figure 4.

### 3.3. Statistical Analysis

Effect sizes (ESs) and confidence intervals (CIs) were calculated to analyze the impact of different exercise modalities from included studies. The results are presented in two separate tables based on the presence of biomechanical misalignment in lower extremity (Table 2 and Table 3). In each table, studies were categorized according to exercise types.

### 3.4. Risk of Bias

**Figure 5:**
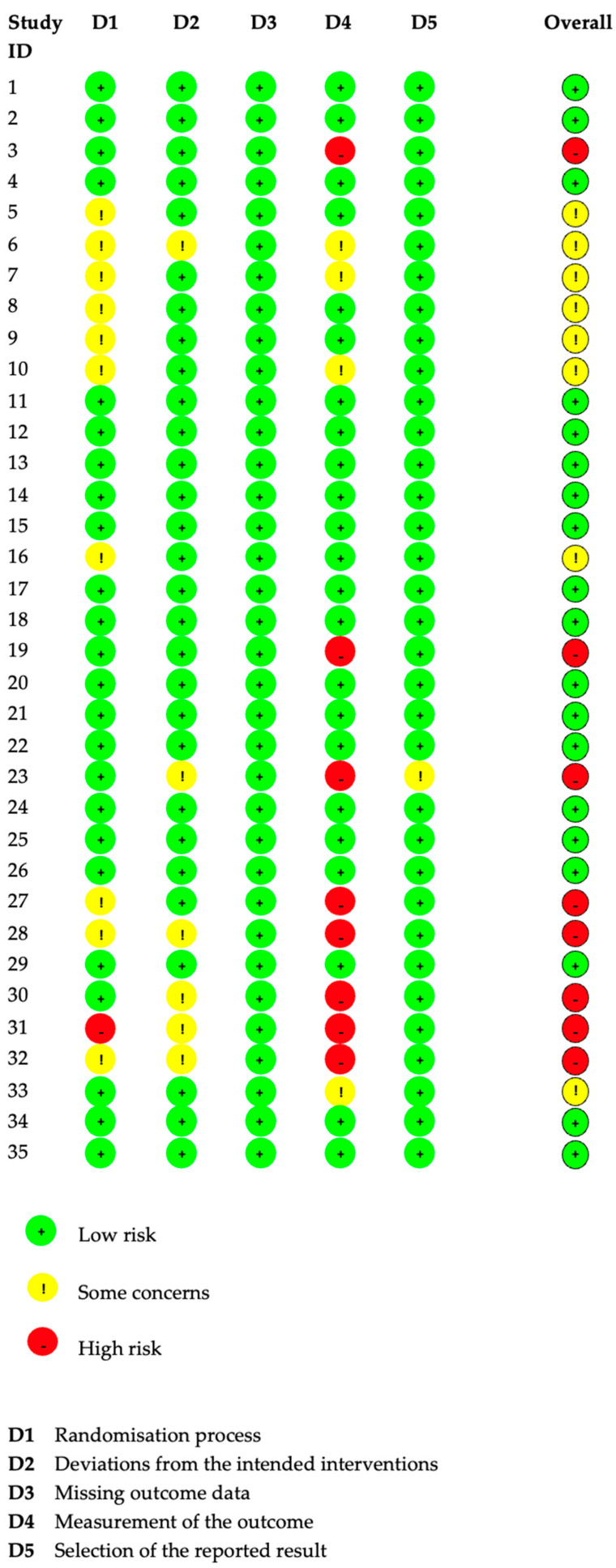
Risk of Bias (RoB2) assessment results.

The quality of the included studies was evaluated with the Cochrane Risk of Bias (RoB) tool. The results of five individual domains and overall bias were shown as low risk, some concerns, and high risk. In evaluating the overall bias, 19 studies showed low risk, 8 studies had some concerns, and 8 studies had a high risk of bias. One of the five domains having some concern or high was shown to be sufficient to affect the overall bias (Figure 5).

## 4. Discussion

In this systematic review, the effects and outcomes of different exercise modalities on frontal plane knee and foot posture in healthy adults were investigated. In order to structure the discussion, studies were categorized into two groups: (1) exercise modalities in healthy adults without any biomechanical misalignment in the lower extremity, which included hip and lower extremity muscle strengthening, core muscle strengthening, foot muscle strengthening, technique training, gait/running retraining, and combined training programs and (2) exercise modalities in healthy adults with a biomechanical misalignment in the lower extremity, such as valgus knee, limited ankle RoM, pronated feet, and flat feet.

### 4.1. Exercise Modalities in Healthy Adults Without a Biomechanical Misalignment in the Lower Extremity

#### 4.1.1. Hip and Lower Extremity Muscle-Strengthening Training

Maintaining a certain level of hip and lower extremity strength is important, particularly for individuals with low muscle strength, to prevent pain and injuries. Surprisingly, in the studies included in this review, hip and lower extremity muscle strengthening programs did not significantly improve knee valgus. Snyder et al. (2009) focused on enhancing the strength of the hip abductor and external rotator muscles in their intervention study [82]. Despite an increase in hip muscle strength after the training, there was no improvement in knee valgus but a significant decrease in foot eversion (*p* = 0.05, *d* = 0.47, CI = [−1.26, 0.32]) (Table 2 and Supplementary Table S1). A previous review [87] supported these findings, indicating that hip-focused neuromuscular training may lead to increased muscle activation but may not always result in biomechanical changes in the lower extremities. Augmented hip muscle strength can enhance proximal stability, improving alignment and mobility in distal joints. However, moderate evidence supports the notion that hip muscle strengthening can influence the knee valgus and lower extremity biomechanics in healthy individuals [41]. Herman et al. (2009) focused on the gluteus medius, gluteus maximus, quadriceps, and hamstring muscles using resistance bands, and McCurdy et al. (2012) included functional exercises such as squats, lunges, step ups, and deadlifts in their training program [66,73]. Both found no significant change in knee valgus following training. However, McCurdy et al. (2012) did not examine hip and knee muscle strength, whereas Herman et al. (2009) did not obtain any changes in knee valgus despite increased lower extremity muscle strength [66,73]. This finding is supported by previous research reporting a weak correlation between lower extremity muscle strength and biomechanics [88].

In two studies, lower extremity muscle strength training was combined with trunk muscle strengthening or upper extremity muscle strengthening. Araujo et al. (2017) combined strengthening exercises for the hip abductors, extensors, external rotators, and lower trunk muscle strengthening [53]. There were no significant changes; however, a downward trend in knee abduction and ankle eversion after the training was observed. On the other hand, Jeong et al. (2020) implemented a program consisting of quadricep and hamstring strengthening in combination with upper trunk muscle-strengthening exercises but did not achieve any changes in knee valgus [69]. It is noteworthy that in some studies, hip and lower extremity strength training does not affect knee valgus but changes ankle eversion. One explanation for this may be that exercises for the external rotator muscles in the studies of Snyder et al. (2009) and Araujo et al. (2017) affected foot position. However, since the other included studies did not measure ankle eversion or foot pronation, it is difficult to compare the effect of these exercises on foot position.

#### 4.1.2. Core Muscles Strengthening Training

Core muscle strengthening is often preferred to stabilize the upper extremities, pelvis, and hips and thus improve lower extremity movements. Furthermore, inadequate core strength can be a risk factor for knee and foot posture abnormalities and injuries [89,90,91].

Two studies implemented a core muscle training program in the present review. In the study by Jeong et al. (2021), the training program consisted of comprehensive core strengthening and additional trunk and lower extremity stretching exercises, while Sasaki et al. (2019) implemented a more basic core strengthening program [70,81]. Jeong et al. (2021) reported that core stabilization training improved muscle activation balance, resulting in an increase in the vastus medialis to vastus lateralis ratio. Although there was no increase in quadriceps and hamstring strength, this increase in ratio significantly reduced knee valgus (*p* = 0.03, *d* = −0.39, CI = [−0.79, 0.02]). Sasaki et al. (2019) found a clinically meaningful decrease in knee valgus during jump landing and a statistically significant decrease during jump landing with large effect sizes (*d* = −1.25, CI = [−2.02, −0.48]); *p* = 0.008, *d* = −1.06, CI = [−1.83, −0.29]). This was also associated with increased hip abductor strength. There was no increase in knee extensor strength, but likely, with the contribution of the Nordic hamstring exercise in the training program, an increase in hamstring strength was observed. This supported the importance of knee flexor/hip extensor strength in knee position, as highlighted in previous research [92]. Additionally, sex differences might be one of the reasons for the varied responses in the strength and activation of different muscle groups after the training programs [93]. Both studies have strongly demonstrated that core exercises reduce knee valgus in individuals without a knee misalignment. However, further studies are needed in our target population to provide sufficient evidence of the effects of core exercises on knee and foot posture.

#### 4.1.3. Foot Muscle-Strengthening Training

There are different exercises to strengthen the foot muscles, which vary depending on the targeted muscle groups. Short foot exercises constitute the prominent exercise modality in the healthy population without impairing foot posture. In Mulligan et al.’s (2013) study, which was conducted on asymptomatic individuals, there was a significant reduction in navicular drop following a short foot exercise program (*p* = 0.01, *d* = −0.31, CI = [−0.92, 0.30]) [75]. The training was more effective in individuals with a higher navicular drop at baseline. However, the practical impact of the intervention may be limited due to the small effect size and wide confidence interval (*d* = −0.31, CI [−0.92, 0.30]).

On the other hand, towel curl exercises target the toe flexor muscles and involve a dynamic movement of dragging the towel under the foot by flexing the toes. Lynn et al. (2012) compared the effects of towel curl and short foot exercises [72]. They found that neither exercise group significantly improved navicular height after the training, but it was reduced in the short foot exercise group. This was parallel to previous findings indicating that towel curl exercises may lead to the dominance of extrinsic toe flexors over the intrinsic foot muscles, as the exercises often recruit the flexor digitorum longus muscle [94]. In Sulowska et al.’s (2016) study, the effects of short foot muscle exercises were compared with reverse tandem gait and Vele’s forward lean training in long-distance runners [83]. In the tandem gait and forward lean group, the aim was to increase the activity of intrinsic foot muscles, as well as to improve appropriate loading and transfer in the lower extremities and feet and enhance neuromuscular control. Both exercise groups showed improvement in foot pronation, but this improvement was slightly greater in the tandem gait and forward lean group. Based on the results of these three studies, we can agree that strengthening the intrinsic muscles of the foot improves foot posture, especially by affecting the position of the navicular, in individuals without malalignment in foot posture.

#### 4.1.4. Technique Trainings

Six studies aimed to improve lower extremity biomechanics and increase movement control and motor skills by improving individuals’ exercise techniques. Two studies used supervised plyometric training with instructions to teach and improve jump and landing techniques in female athletes. Baldon et al. (2013) found a significant reduction in knee abduction after 8 weeks of training (*p* = 0.01, *d* = −1.24, CI = [−1.78, −0.69]) [59]. Herrington (2010) achieved an improvement in knee valgus in a shorter period (4 weeks) (*p* = 0.002) [67]. However, it is difficult to interpret the practical significance and reliability of the Herrington et al. (2010) study, since missing group means and standard deviations prevent the calculation of effect size and confidence intervals. Nevertheless, these findings confirm that plyometric training enhances lower extremity strength, stability, and proprioception [34].

As for the effects of feedback types during jumping training, only one study investigated the effect of different feedbacks on knee abduction in training programs involving strength, plyometric, and balance exercises. Ghanati et al. (2022) compared differential learning (DL), self-controlled feedback (SF), and external focus (EF) of attention on knee abduction angle in male athletes [63]. Both DL and EF approaches significantly reduced knee abduction angle with large effects sizes (*p* = 0.001, *d* = −1.69, CI = [−2.42, −0.96]); *p* = 0.001, *d* = −1.18, CI = [−1.88, −0.49]), with DL showing slightly superior performance (*p* = 0.001), indicating the potential benefits of incorporating variability in training. In future studies, examining the effect of different types of feedback in the same type of training or implementing the same type of feedback during different types of training may provide us with more detailed insights into the effect of feedback.

Another type of technique training is to control the alignment of the lower limbs during functional exercises (squats, lunges, etc.) with verbal and visual feedback, and we identified two studies that practiced this. Kato et al. (2008) found a significant improvement in knee abduction after training the individuals to control the lower extremity alignment during squats, lunges, jump landing, and balance exercises (*p* < 0.05, *d* = −0.69, CI = [−1.42, 0.04]) [71]. In Dawson et al.’s study (2015), hip muscle strengthening and skill training were compared to see the effects on frontal plane projection angle (FPPA) [58]. Although both training groups showed a significant improvement in FPPA, this was greater in the skill training group (*p* = 0.001, *d* = −1.64, CI = [−2.41, −0.87]; *p* = 0.003, *d* = −1.61, CI = [−2,43, −0.79]). This is, however, a surprising result in terms of the previously discussed effect of hip strengthening training on knee frontal plane posture. These two studies focused on how extremities should be positioned during exercises. This was consistent with previous findings, which indicated that using internal focus for learning a technique and having cognitive control of the extremities, and subsequently shifting internal and external focus, enhances the quality of the training [95,96]. An interesting result was that, following the training, the improvement in knee posture occurred independently of muscle strength, showing the effectiveness of the training in sensorimotor learning. In a different design of technique training. Herman et al. (2008) compared video feedback-assisted stop-jump landing training alone with a lower extremity strength training program [65]. No improvement in knee valgus was seen in either the strength training group or the feedback-assisted landing training group combined with strengthening.

Jumping and functional exercises, such as squats and lunges, enhance lower extremity biomechanics. These activities are effective for developing proper technique and maintaining alignment of the lower extremities, while also contributing to increased strength and stability in this region. However, future studies are needed to investigate the effects of feedback to enhance the effectiveness of technique training and offer more personalized training programs.

#### 4.1.5. Gait/Running Retraining

Two studies implemented running retraining programs to enhance running techniques to reduce foot pronation or eversion in recreational runners. Da Silva Neto et al. (2022) aimed to reduce the vertical ground reaction force by asking the participants to run more smoothly with a combination of static balance training to control plantar loading and distribute the load on the plantar area [57]. The foot pronation (FPI-6) significantly decreased after the 2-week intervention (*p* = 0.02, *d* = −0.31, CI = [−0.97, 0.36]). However, results differed in the study of Dunn et al. (2018) [61]. They implemented pose running retraining with verbal and video feedback to improve participants’ perception of falling and pulling in running. There was no change in ankle eversion as a result of training. Nevertheless, there were some differences in these studies. For example, Dunn et al.’s (2018) training program lasted only three sessions, and they measured ankle eversion using 3D motion analysis during running. In these two studies, real-time visual feedback during gait retraining focusing on both the extremities and the body (internal), as well as the movement (external), was provided. Previous reviews have also confirmed that this intervention positively affects lower extremity mechanics in runners [97], healthy individuals, and individuals with knee or hip osteoarthritis [98,99]. Gait retraining is also a form of technique training, and we believe that it can lead to positive outcomes, particularly in foot positioning. However, studies within our target population are limited. The two studies included in this review were conducted on runners, and the results may differ in individuals with less experience in running or walking. Additionally, we recommend further research on gait retraining and weight transfer training in individuals with foot posture abnormalities.

#### 4.1.6. Combined Trainings

Some studies have implemented multimodal training programs to alter lower extremity posture. The idea that combining various types of exercises to create a training program positively impacts lower extremity biomechanics is not always accurate. In two studies, combined exercises have been used to improve core and lower extremity strength, endurance, balance, stabilization, and motor control. Baldon et al. (2014) implemented a training program aimed at improving proper lower extremity alignment during dynamic activities with verbal feedback in addition to exercises targeting core, hip abductor, and external rotator strength and stabilization and balance exercises for female recreational athletes [60]. This study showed a statistically and clinically significant decrease in knee abduction with a large effect size (*p* = 0.001, *d* = −1.17, CI = [−1.78, −0.55]), in addition to increased hip eccentric abductor and external rotator strength.

On the other hand, Chappell et al. (2008) investigated the effect of a neuromuscular training program combining core strengthening, dynamic joint stability, balance, jumping, and plyometric training on knee valgus in female athletes [56]. A surprising outcome was that while there was no change in knee valgus during the drop jump task in the intervention group after training, there was a reduction in knee valgus during the stop jump task. This highlights how dependent the post-intervention results can be on the measurement task. In this context, expertise is crucial when interpreting results in interventional studies. Our perspective on combined exercises is that more exercises do not necessarily lead to better results. This approach may be time-consuming and challenging to maintain exercise adherence and motivation.

### 4.2. Exercise Modalities in Healthy Adults with a Biomechanical Misalignment

#### 4.2.1. Valgus Knee

We determined that feedback-based jumping training has different effects on individuals with increased knee valgus than those without. In Tate et al.’s (2013) study, participants attempted to control the knee position during jump landing by increasing external focus with the help of a mirror [84]. Ericksen et al. (2016) compared the effects of traditional feedback (internal focus) with real-time feedback (external focus) during jump landing [62]. Both studies could not find any decrease in knee abduction in female athletes after the training. Nevertheless, we are unable to clarify the reasons behind the differing outcomes in knee valgus observed in individuals with increased knee valgus. In contrast, we identified that the three studies focusing on controlling lower extremity position in this population improved frontal plane knee posture. Mozafaripour et al. (2022) and Olson et al. (2011) found a reduction in knee valgus and FPPA after the training programs focused on lower extremity neuromuscular training and maintaining proper alignment in the lower extremities (*p* = 0.001, *d* = −1.31, CI = [−2.00, 0.62]; *p* = 0.001, *d* = −1.13, CI = [−1.88, 0.38]) (Table 3 and Supplementary Table S2) [74,77]. Similarly, in Palmer et al.’s (2015) study, there was a reduction in knee valgus in the functional motor control training group, which focused on controlling knee position during squats, approximately 10°, and in the hip abductor strengthening group, approximately 5° [79]. However, these reductions are not statistically significant and had a low effect size during single leg squat (*d* = −0.25, CI = [−0.82, 0.31]; *d* = −0.58, CI = [−1.17, 0.00]) and single leg landing (*d* = −0.08, CI = [−0.64, 0.48]; *d* = −0.14, CI = −0.73, 0.44]) for both interventions. Additionally, no superiority was found between the two exercise groups.

In another study, Bell et al. (2013) found significant improvements in knee valgus (*p* = 0.001, *d* = −0.61, CI = [−1.16, −0.07]) and medial knee displacement (*p* = 0.02, *d* = −1.45, CI = [−2.00, −0.91]) after hip and ankle RoM and strengthening exercises [54]. While hip-strengthening programs did not show a significant effect in individuals without knee valgus, this study found that hip strengthening improved knee posture in individuals with knee valgus. However, since this is the only study focused on hip and ankle strengthening in healthy individuals with knee valgus in the present review, further evidence is needed.

A key difference we observed in studies conducted on individuals with increased knee valgus is that hip and lower extremity strength programs, especially when combined with controlling lower extremity alignment during these exercises, reduced knee valgus. This could be because increased knee valgus may result from decreased strength and neuromuscular control, making these exercises effective in improving knee posture.

#### 4.2.2. Limited Ankle RoM

Limited ankle dorsiflexion might cause the knee medial placement, but Howe et al.’s (2022) findings did not support this hypothesis [68]. They demonstrated that although ankle mobility exercises increased the ankle joint’s range of motion in individuals with limited ankle dorsiflexion after the training when combined with lower extremity strength exercises (such as squats, lunges, deadlifts, and jumps), this did not lead to changes in knee frontal plane projection angle. However, unlike in Bell et al.’s (2013) study, participants did not demonstrate increased knee valgus; therefore, it was not clear whether the training was effective in improving knee posture [54].

#### 4.2.3. Pronated Feet

We identified four studies that examined how exercises influence foot pronation in healthy individuals with pronated feet.

Goo et al. (2016) compared the effects of combining gluteal maximus and foot intrinsic muscles strengthening with foot intrinsic foot muscles strengthening alone on navicular drop [64]. Both interventions improved foot posture, but the combined training group decreased navicular drop significantly with a very large effect size (*p* < 0.05, *d* = −3.9, CI = [−4.22, −2.77]). Similarly, Alam et al. (2019) demonstrated that adding tibialis posterior strengthening and iliopsoas stretching to towel curl exercises had superior effects on foot posture than towel curl exercises alone (*d* = −1.90), and this significantly reduced navicular drop (*p* = 0.001, *d* = −2.78, CI = [−3.36, −2.19]) [52]. Sanchez et al. (2020) determined that exercises targeting the foot’s intrinsic and extrinsic muscles and core muscle strengthening significantly reduced foot pronation (*p* = 0.001, *d* = −0.88, CI = [−1.39, −0.37]) [80]. The movements of the hip influence foot pronation and, consequently, the femur and tibia. These findings demonstrate that alterations in proximal structures can affect foot positioning. Therefore, exercises focusing on the hip and trunk may be beneficial for individuals with foot pronation.

The effect of short foot exercises on pronated feet was not clear. Carrasco et al. (2020) found a tendency of the foot to be in a neutral position after short foot exercises, but this was insufficient to prove the effectiveness of the intervention since this was observed in both the control and intervention groups [78].

#### 4.2.4. Flat Feet

Flat feet are characterized by a lower medial arch and, consequently, a flattening of the foot’s sole. Therefore, strengthening the intrinsic foot muscles is important for the muscles of the foot’s arch in individuals with flat feet, as this can alter the position of the foot’s sole and potentially affect pronation. The included studies show that short foot exercises effectively reduce foot pronation and improve navicular and medial arch height in individuals with flat feet. These results are consistent with the findings from the previous review [100]. Okamura et al. (2020) found a significant decrease in FPI (*p* = 0.01, *d* = −0.68, CI = [−1.37, 0.01]), whereas Unver et al. (2019) found a significant decrease in FPI (*d* = 0.001, *d* = −1.12, CI = [−3.48, 1.24]) and navicular drop (*p* = 0.001, *d* = 0.88, CI = [−1.72, −0.04]) following a short foot exercise training [76,85]. However, there was also a significant decrease in the FPI total score in the control group in Okamura et al.’s study (*p* = 0.02, *d* = −0.64, CI = [−1.33, 0.04]).

In the study by Utsahachant et al. (2023), both lower extremity strength exercises combined with short foot exercises and short foot exercises alone exhibited comparable and beneficial effects in decreasing navicular drop (*p* = 0.001, *d* = −1.15, CI = [−1.78, −0.52]; *p* = 0.001, *d* = −1.58, CI = [−2.21, −0.95]) [86]. Brijwasi et al. (2023) showed that short foot exercises and gluteal muscle strengthening reduced navicular drop (*p* = N/A, *d* = −1.96, CI = [−2.07, −1.86]) and increased medial longitudinal arch height (*p* = N/A, *d* = 2.91, CI = [0.22, 5.61]) [55].

### 4.3. Effects of the Participant, Measurement Method, and Implementation of the Training Programs

#### 4.3.1. Participants

The present review only included studies conducted on healthy individuals. The effects of exercise may differ depending on whether the individual is healthy or experiencing injury or pain. In cases where pain is a factor, biomechanical alignment may also improve when pain reduces after training. However, since individuals with pain and injury may demonstrate a higher incidence of knee and foot displacement than healthy individuals, the effects of interventions in these populations may be greater.

Furthermore, 13 studies in the present review involved participants who were healthy but exhibited increased knee valgus, foot pronation, or flat feet. In 10 out of 13 studies, training programs significantly improved lower extremity posture.

Significant improvements were observed in 14 out of 22 studies in individuals without biomechanical malalignments. This suggested that individuals with existing postural abnormalities in the knee or foot were more likely to benefit from exercise. In such a case, identifying the specific needs of these individuals may facilitate a more targeted exercise program with more noticeable outcomes.

Participants in different studies had different levels of physical activity. Variations in participants’ physical activity levels across different studies may complicate the interpretation of pre-exercise fitness and post-exercise gains on biomechanical parameters. Studies involving individuals with lower physical activity levels may provide more precise insights into strength, fitness, and functional skill improvements. Since there are also physiological, anatomical, and biomechanical differences between males and females, normative values and improvements must be evaluated according to gender [101].

#### 4.3.2. Measurement Method

In the included studies, measurement tasks involved various types of movements. When the training program aligns with the measurement task (e.g., a jumping training program and jump landing task), individuals might perform better biomechanical control during measurement due to repeated practice. However, it is crucial to note that results may differ in other tasks for the same individual. Therefore, in the analysis, evaluating both the measurement task and individual conditions is essential for making accurate inferences about the impact of interventions.

This consideration extends to the measurement method or tool as well. Some studies measured lower extremity posture during dynamic tasks, while others focused on static positions. Since dynamic activities may trigger more knee valgus, improvements observed during squatting may not be evident in a standing position. Similarly, 3D and 2D measurements may provide different improvements in knee valgus and knee abduction after an intervention. Foot pronation and eversion have also been measured during different movements using various methods such as 3D analysis, navicular and medial longitudinal arch height, and the foot posture index. Improvements in static and more subjective measurements like the navicular drop test and foot posture index may differ from kinematic analyses. However, no superiority of either 2D or 3D measurements has been identified in determining the effectiveness of exercise interventions in the included studies. Thus, interpreting complex terminology is important at this point. For instance, improvements in navicular height may not necessarily correlate with changes in foot pronation or calcaneal eversion, and vice versa. Progress in one task or measurement may not be evident in the other.

#### 4.3.3. Implementation of the Training Program

We believe that a sufficient minimum training length is necessary to achieve muscle strength gains, neuromuscular adaptations, and technical improvements. However, structural changes such as knee and foot posture adaptations may require longer intervention periods. The training programs of the included studies ranged from 1 week to 10 weeks. The shortest intervention length among the included studies was 1 week, and none of these studies reported statistically significant improvements within this timeframe. The minimum length required to obtain meaningful biomechanical improvements from exercise interventions appears to be 3–4 weeks. However, there were heterogeneous findings in studies implementing the trainings over 3 weeks. Thus, precisely determining the optimal length of the training program was challenging. The results obtained from the studies were independent of the length of the training. Additionally, the optimal training length may vary depending on the type of exercise. Therefore, the length and frequency of the training program should be tailored to the type of training. Future studies should explore the minimum length for different exercise modalities to optimize intervention efficiency.

Expected results may vary among individuals and are directly linked to their adaptation to exercise. As mentioned earlier, the participant’s profile plays a role in this. An individual’s physical capacity, biomechanical characteristics, and anatomical structure may be decisive in determining the time needed to see the effects of the training program. Therefore, it is essential to customize exercise programs based on individual needs.

##### Limitations

The present review has some limitations. First, it only included studies conducted in healthy adults, thus not representing the results of studies in those with injuries or pain and younger or older individuals. Similarly, some studies had non-athlete participants alongside athletes, which could have influenced the outcomes. Additionally, studies with participants with foot and knee postural abnormalities were included, leading to varied results. Although we defined outcome parameters clearly in our literature search, comparing and interpreting the results was challenging due to differences in measurement methods. We also aimed to examine studies focusing on both knee and foot frontal plane biomechanics. However, due to the limited number of studies, the effects of exercises on both joints and their interaction did not yield clear results. Not all studies in each exercise category were found to be consistent. Therefore, it did not provide sufficient evidence regarding the effectiveness of some exercise modalities. While previous reviews have focused on the effects of a single type of exercise on the lower extremities, our review has been more comprehensive regarding exercise modalities. Finally, the present review did not include a meta-analysis.

## 5. Conclusions

In this systematic review, we analyzed the effects of different exercise modalities on frontal plane knee and foot posture in healthy adults.

First, skill and technique training with a focus on controlling lower extremity alignment was the most frequently implemented intervention in healthy individuals without a biomechanical misalignment in the lower extremities, and a substantial number of studies reported significant improvements in knee valgus. Second, core muscle strengthening strongly improved knee valgus in healthy populations. In contrast, hip and lower extremity strengthening were insufficient to improve knee valgus. Foot muscle strengthening, combined training programs, and gait/running retraining, which aimed to enhance foot pronation in individuals without a biomechanical misalignment, showed inconsistent effects. However, training that focused on hip, core and foot muscles strength decreased foot pronation in individuals with pronated feet and strength training for foot intrinsic muscles alone or in combination with lower extremity strength training and improved foot posture in individuals with flat feet.

It has to be acknowledged that in some intervention strategies the number of studies is rather low, which limits the general validity of the evidence.

Nevertheless, among all studies analyzed in the present review, technique training and core muscle strengthening were the most effective exercise modalities for improving knee posture, whereas foot, hip, and core muscle strengthening played a key role in enhancing foot posture in healthy individuals.

Based on our findings, studies on healthy adults with lower extremity biomechanical misalignment are limited. There is a need for further research on preventive exercise programs to address biomechanical risk factors and minimizing pain and injury risk. On the other hand, future research on individuals without misalignment may contribute to improving quality of life, enhancing sports performance, and promoting physical activity. Therefore, future research should explore how preventive exercise programs in healthy individuals contribute to maintaining health and improving quality of life, and how they should differ from rehabilitative exercises and treatments implemented in symptomatic individuals.

## Figures and Tables

**Table 1:** Study characteristics.

Author’s Name	Participants	Groups	Measurement Tool	Measurement Task	Intervention	Outcome Parameters	Results
**Alam et al., 2019** [52]	collegiate students with bilateral pronated feet (*n* = 28) (age 18–25 years)	2 intervention groups	navicular drop test	seating/standing	TP strengthening and IP stretching (I1) (3 × /6 weeks) towel curl exercises (I2) (7 × /6 weeks)	navicular drop	greater improvement for navicular drop in I1 than I2 group
**Araujo et al., 2017** [53]	healthy females (*n* = 36) (age 18–35 years)	1 intervention, 1 control group	motion capture system	step down	hip and trunk strengthening exercises (3 × /8 weeks)	knee abduction, foot eversion angle	no significant changes in knee abduction and foot eversion
**Bell et al., 2013** [54]	healthy males and females with visually identified knee valgus (*n* = 32) (age 18–35 years)	1 intervention, 1 control group	motion capture system	double leg squat	hip- and ankle-based strength training program (3 × /3 weeks)	knee valgus angle, medial knee displacement	significant reduction in knee valgus angle and medial knee displacement
**Brijwasi et al., 2023 ** [55]	healthy males and females with flexible flat foot (*n*= 49) (age 20–22 years)	1 intervention, 1 control group	navicular drop test goniometer	standing	ankle dorsi-plantarflexion, short foot exercises, gluteal muscle strengthening, and calf stretches (3 × /6 weeks)	navicular drop, medial longitudinal arch angle	significant reduction in navicular drop and increase in medial longitudinal arch angle in training group
**Chappell et al., 2008 ** [56]	healthy female collegiate athletes (*n* = 30) (age 18–21 years)	1 intervention group	motion capture system	drop jump and vertical stop jump	neuromuscular training program (6 × /6 weeks)	knee valgus angle	no significant change in knee valgus after the intervention
**Da Silva Neto et al., 2022** [57]	recreational runners (*n* = 24) (age 20–45 years)	1 intervention, 1 control group	foot posture index (FPI-6)	standing	gait retraining and static balance training with visual biofeedback (4 × /2 weeks)	foot pronation	significant reduction in foot pronation
**Dawson et al., 2015** [58]	recreationally active males and females (*n* = 17) (age 24–36 years)	2 intervention groups	digital camera	single leg squat	hip strengthening training (I1), skill acquisition training (I2) (3 × /6 weeks)	knee frontal plane projection angle (FPPA)	significant reduction in FPPA in both groups
**De Marche Baldon et al., 2013** [59]	healthy recreational female athletes (*n* = 36) (age 18–22 years)	1 intervention, 1 control group	digital cameras	single leg squat	plyometric training (3 × /8 weeks)	knee abduction angle	significant reduction in knee abduction in the intervention group
**De Marche Baldon et al., 2014** [60]	healthy recreational female athletes (*n*= 28) (age 18–22 years)	1 intervention, 1 control group	digital cameras	single leg squat	functional stabilization training (3 × /8 weeks)	knee abduction angle	significant reduction in knee abduction in the intervention group
**Dunn et al., 2018** [61]	recreational runners (*n* = 20) (age 26–33 years)	1 intervention, 1 control group	motion capture system	running	pose running retraining (2 h × 3 sessions)	foot eversion angle	no significant change in peak foot eversion angle
**Ericksen et al., 2016 ** [62]	recreationally active females with dynamic knee valgus (*n* = 48) (age 18–30 years)	2 intervention groups, 1 control group	motion capture system	jump landing	traditional feedback jump landing training and real time feedback jump landing training (I1), traditional feedback jump landing training (I2) (3 × /4 weeks)	knee abduction angle	no significant change in knee abduction angle for all groups
**Ghanati et al., 2022** [63]	male athletes (*n* = 48) (age 20–25 years)	3 intervention groups, 1 control group	motion capture system	single leg vertical drop jump	squat and jump trainings with self-controlled feedback (SF) (I1), external focus (EF) of attention (I2), and differential learning (DL) strategies (I3) (2 × /8 weeks)	knee abduction angle	significant reduction in knee abduction for DL and EF groups
**Goo et al., 2016** [64]	healthy males and females with pronated feet (*n*= 18) (age 20–23 years)	2 intervention groups	navicular drop test	standing	gluteus maximus muscle and intrinsic foot muscle strengthening (I1), intrinsic foot muscle strengthening (I2) (5 × /4 weeks)	navicular drop	significant reduction in navicular drop in the combined training group
**Herman et al., 2009** [65]	female recreational athletes (*n* = 66) (age 18–30 years)	1 intervention, 1 control group)	motion capture system	stop-jump	lower extremity strength training (3 × /9 weeks)	knee valgus angle	no significant change in knee valgus
**Herman et al., 2008** [66]	female recreational athletes (*n* = 58) (age 18–30 years)	1 intervention, 1 control group	motion capture system	stop-jump	strength training with video-assisted feedback (3 × /9 weeks)	knee valgus angle	no significant change in knee valgus for both group
**Herrington., 2010** [67]	national female basketball players (*n* = 15) (age 18–22 years)	1 intervention group	digital camera	drop jump task, jump-shot task	progressive jump training (3 × /4 weeks)	knee valgus angle	significant reduction in knee valgus
**Howe et al., 2022** [68]	healthy females and males with restricted ankle dorsiflexion range of motion (*n* = 11) (age 20–22 years)	2 intervention groups	digital camera	bilateral drop landing	lower extremity strength and ankle mobility training (I1), lower extremity strength training (I2) (3 × /4 weeks)	knee frontal plane projection angle (FPPA)	no significant change in FPPA
**Jeong et al., 2020** [69]	recreationally active females (*n* = 25) (age 22–26 years)	1 intervention, 1 control group	motion capture system	side-step cutting	lower extremity muscle strength training (3 × /10 weeks)	knee valgus angle	no significant reduction in knee valgus
**Jeong et al., 2021** [70]	recreationally active men (*n* = 48) (age 19–25 years)	1 intervention, 1 control group	motion capture system	side-step cutting	core muscle strengthening (3 × /10 weeks)	knee valgus angle	significant decrease in knee valgus in intervention group
**Kato et al., 2008** [71]	female college basketball players (*n* = 20) (age 19–21 years)	1 intervention, 1 control group	digital camera	quick stop-jump task	lower extremity alignment control training (3 × /4 weeks)	knee abduction angle	significant reduction in knee abduction in the intervention group
**Lynn et al., 2012** [72]	healthy males and females (*n* = 24) (age 20–25 years)	2 intervention groups, 1 control group	standing navicular height	standing	short foot exercises (I1), towel curl exercises (I2) (7 × /4 weeks)	navicular height	no significant change in navicular height for all groups
**McCurdy et al., 2012** [73]	recreationally active, healthy females (*n* = 27) (age 19–23 years)	1 intervention, 1 control group	motion capture system	unilateral and bilateral drop jump	lower extremity weight-bearing resistance training (2 × /8 weeks)	knee valgus angle	no significant change in knee valgus for both groups
**Mozafaripour et al., 2022** [74]	healthy males with dynamic knee valgus (*n*= 30) (age 18–28 years)	1 interventio, 1 control group	motion capture system	single leg squat	comprehensive corrective exercises for hip, leg, and foot (3 × /8 weeks)	knee valgus angle	significant improvement in knee valgus angle in the intervention group
**Mulligan et al., 2013** [75]	healthy females and males (*n* = 21) (age 22–30 years)	1 intervention group	navicular drop test	standing	short foot exercises (7 × /4 weeks)	navicular drop	significant reduction in navicular drop at 4-week and 8-week follow-up after intervention
**Okamura et al., 2020** [76]	healthy females and males with pes planus (*n* = 20) (age 19–22 years)	1 intervention, 1 control group	foot posture index (FPI-6), navicular drop, test motion capture system	standing and walking	short foot exercises (3 × /8 weeks)	foot pronation, navicular drop	significant improvement of FPI score in both groups, significant reduction in navicular drop in the control group
**Olson et al., 2011** [77]	healthy females with medial knee placement (*n* = 18) (age 18–25 years)	1 intervention group	digital camera, motion capture system	single leg step down	neuromuscular training (3 × /4 weeks)	knee frontal plane projection angle (FPPA), knee abduction angle	significant reduction in FPPA, but no change in 3D knee abduction
**Pabon-Carrasco et al., 2020** [78]	healthy females and males with pronated foot (*n* = 85) (age 19–22 years)	1 intervention, 1 control group	navicular drop test, foot posture index (FPI-6)	standing	short foot exercises (7 × /4 weeks)	navicular drop, foot pronation	no significant change in foot pronation and navicular drop for both groups
**Palmer et al., 2015** [79]	military personnel volunteers with increased dynamic knee valgus and internal rotation (*n* = 29) (age 29–39 years)	2 intervention groups	motion capture system	single leg squat and single leg landing	isolated hip strengthening (I1), functional motor control exercises (I2) (3–4 × /5 weeks)	knee valgus angle	no statistically significant improvement in knee valgus for both groups
**Sanchez-Rodriguez et al., 2020** [80]	healthy females and males with pronated feet (*n* = 36) (age 18–27 years)	1 intervention, 1 control group	foot posture index (FPI-6)	standing	foot intrinsic and extrinsic muscle and core muscle-strengthening exercises (2 × /9 weeks)	foot pronation	significant reduction in foot pronation in the intervention group
**Sasaki et al., 2019** [81]	female collegiate basketball players (*n* = 17) (age 18–22 years)	1 intervention, 1 control group	motion capture system	jump landing and single leg squat	core strength training (4 × /8 weeks)	knee valgus angle	significant reduction in knee valgus angle during single leg squat in the intervention group
**Snyder et al., 2009** [82]	healthy females (*n* = 13) (age 20–23 years)	1 intervention group	motion capture system	running	closed-chain hip rotation and abduction strengthening exercises (3 × /6 weeks)	knee abduction, foot eversion angle	significant reduction in foot eversion
**Sulowska et al., 2016** [83]	long distance runners (*n* = 25) (age 22–35 years)	2 intervention groups	foot posture index (FPI-6)	standing	Vele’s forward lean and reverse tandem gait (I1), short foot exercises (I2) (7 × /6 weeks)	foot pronation	no significant change for both group in total score of FPI
**Tate et al., 2013** [84]	female recreational athletes with medial knee displacement during jump landing (*n* = 26) (age 18–30 years)	1 intervention, 1 control group	motion capture system	counter movement jump	supervised and homebased counter movement jump training (3 sessions/1 week)	knee abduction angle	no significant change in knee abduction
**Unver et al., 2020 ** [85]	healthy males and females with pes planus (*n*= 41) (age 18–25 years)	1 intervention, 1 control group	navicular drop test, foot posture index (FPI-6)	standing	short foot exercises (7 × /6 weeks)	navicular drop, foot pronation	significant reduction in navicular drop and FPI score
**Utsahachant et al., 2023** [86]	healthy females and males with flexible flat foot (*n* = 45) (age 20–29 years)	2 intervention groups, 1 control group	navicular drop test, motion capture system	standing and walking	short foot exercises (I1), lower extremity strengthening and short foot exercises (I2) (3 × /6 weeks)	navicular drop	significant reduction in navicular drop for both intervention group

I1, I2, I3: Intervention Groups 1, 2, and 3, respectively.

**Table 2:** Effect sizes (ESs) and confidence intervals (CIs) of studies in individuals without a biomechanical misalignment in lower extremity.

Author’s Name	Parameter	Sample Size	Number of Groups	ES (Between Groups)	CI 95% (Between Groups)	ES (Within Groups)	CI 95% (Within Groups)
**Hip and Lower Extremity Muscle-Strengthening Training**							
**Snyder et al., 2009 ** [82]	Knee Abduction Angle	13	1	N/A	N/A	I: 0.19	I: [−0.58, 0.96]
	Foot Eversion Angle	13	1	N/A	N/A	I: −0.47	I: [−1.26, 0.32]
**Herman et al., 2008** [66]	Knee Valgus Angle	66	2	−0.08 (d)	[−0.29, 0.20]	I: 0.016 C: 0.096	I: [−0.39, 0.42] C: [−0.31, 0.50]
**McCurdy et al., 2012** [73]	Knee Valgus Angle (Unilateral Drop Jump)	20	2	−0.87 (d)	[−0.94, 0.07]	I: −0.40 C: 0.46	I: [−1.13, 0.33] C: [−0.26, 1.19]
	Knee Valgus Angle (Bilateral Drop Jump)	20	2	0.13 (d)	[−0.44, 0.57]	I: 0.42 C: 0.31	I: [−0.31, 1.14] C: [−0.41, 1.04]
**Araujo et al., 2017** [53]	Knee Abduction Angle	34	2	−0.37 (d)	[−0.53, 0.16]	I: 0.072 C: 0.505	I: [−0.49, 0.63] C: [−0.05, 1.06]
	Foot Eversion	34	2	0.35 (d)	[−0.18, 0.52]	I: 0.064 C: −0.264	I.: [−0.50, 0.62] C.: [−0.82, 0.30]
**Jeong et al., 2020** [69]	Knee Valgus Angle	25	2	−0.07 (d)	[−0.45, 0.38]	I: 0.17 C: 0.70	I: [−0.49, 0.84] C: [−0.38, 0.89]
**Core Muscle Strengthening**							
**Jeong et al., 2021** [70]	Knee Valgus Angle	48	2	−0.50 (d)	[−0.56, 0.06]	I: −0.39 C: 0.17	I: [−0.79, 0.02] C: [−0.41, 0.74]
**Sasaki et al., 2019** [81]	Knee Valgus Angle (Jump Landing)	17	2	−1.44 (d)	[−1.24, −0.21]	I: −1.25 C: 0.36	I: [−2.02, −0.48] C: [−0.46, 1.17]
	Knee Valgus Angle (Single Leg Squat)	17	2	−1.31 (d)	[−1.17, −0.14]	I: −1.06 C: 0.21	I: [−1.83, −0.29] C: [−0.60, 1.03]
**Foot Muscle Strengthening**							
**Mulligan et al., 2013** [75]	Navicular Drop	21	1	N/A	N/A	I: −0.31	I: [−0.92, 0.30]
**Lynn et al., 2012** [72]	Navicular Height	24	3	0.13 (f)	[0.0, 0.76]	I1: −0.44 I2: 0.0 C: −0.07	I: [−1.26, 0.37] I: [−0.82, 0.82] C: [−0.88, 0.75]
**Sulowska et al., 2016** [83]	Foot Pronation	25	2	−0.08 (d)	[−0.47, 0.38]	I1: −0.51 I2: −0.44	I1: [−1.15, 0.13] I2: [−1.11, 0.22]
**Technique Training**							
**De Marche Baldon et al., 2013** [59]	Knee Abduction Angle	36	2	0.65 (d)	[−0.02, 0.68]	I: −1.24 C: 0.03	I: [−1.78, −0.69] C: [−0.51, 0.58]
**Herrington et al., 2010** [67]	Knee Valgus Angle (Drop Jump, Jump Shot Task)	15	1	N/A	N/A	N/A	N/A
**Ghanati et al., 2022** [63]	Knee Abduction Angle	42	4	0.94 (f)	[0.67, 1.14]	I1: −0.49 I2: −1.18 I3: −1.69 C: 0.27	I1: [−1.22, 0.24] I2: [−1.88, −0.49] I3: [−2.42, −0.96] C: [−0.43, 0.96]
**Kato et al., 2008** [71]	Knee Abduction Angle	20	2	−0.44 (d)	[−0.73, 0.28]	I: −0.69 C: −0.17	I: [−1.42, 0.04] C: [−0.90, 0.56]
**Dawson et al., 2015** [58]	Knee Frontal Plane Projection Angle	17	2	−0.07 (d)	[−0.60, 0.52]	I1: −1.64 I2: −1.61	I1: [−2.41, −0.87] I2: [−2.43, −0.79]
**Herman et al., 2009** [65]	Knee Valgus Angle	58	2	−0.28 (d)	[−0.41, 0.13]	I1: −0.15 I2: 0.13	I1: [−0.57, 0.28] I2: [−0.29, 0.56]
**Gait/running Retraining**							
**Da Silva Neto et al., 2022** [57]	Foot Posture Index (Right Foot)	24	2	−0.31 (d)	[−0.60, 0.29]	I: −0.31 C: 0.0	I: [−0.97, 0.36] C: [−0.67, 0.67]
	Foot Posture Index (Left Foot)	24	2	−0.39 (d)	[−0.63, 0.24]	I: −0.39 C: 0.0	I: [−1.06, 0.28] C: [−0.67, 0.67]
**Dunn et al., 2018** [61]	Foot Eversion Angle	20	2	0.66 (d)	[−0.18, 0.84]	I: 0.18 C: −0.50	I: [−0.55, 0.91] C: [−1.23, 0.23]
**Combined Trainings**							
**De Marche Baldon et al., 2014** [60]	Knee Abduction Angle	28	2	1.26 (d)	[0.22, 1.04]	I: −1.17 C: 0.23	I: [−1.78, −0.55] C: [−0.38, 0.85]
**Chappell et al., 2008** [56]	Knee Abduction Angle (Drop Jump)	30	1	N/A	N/A	I: −0.12	I: [−0.62, 0.39]
	Knee Abduction Angle (Stop Jump)	30	1	N/A	N/A	I: −0.17	I: [−0.67, 0.34]

SD: Standard deviation; CI: confidence interval; ES: effect size; N/A: not applicable; I: intervention group; I1, I2, I3: intervention groups 1, 2, and 3, respectively; C: control group; d: Cohen’s d effect size; d: 0.2 ≤ d < 0.5: small effect size; 0.5 ≤ d < 0.8: medium effect size; d ≥ 0.8: large effect size; f: Cohen’s f effect size; 0.1 ≤ f < 0.25: small effect size, 0.25 ≤ f < 0.4: medium effect size; f ≥ 0.4: large effect size.

**Table 3:** Effect sizes (ESs) and confidence intervals (CIs) of studies in individuals with a biomechanical misalignment in lower extremity.

Author’s Name	Parameter	Sample Size	Number of Groups	ES (Between Groups)	CI 95% (Between Groups)	ES (Within Groups)	CI 95% (Within Groups)
**Valgus knee**							
**Technique trainings**							
**Tate et al., 2013** [84]	Knee abduction angle	26	2	0.28 (d)	[−0.28, 0.55]	I: −0.04 C: −0.34	I: [−0.65, 0.56] C: [−0.94, 0.27]
**Ericksen et al., 2016** [62]	Knee abduction angle	26	3	0.27 (f)	[0.0, 0.67]	I1: 0.66 I2: 0.42 C: 0.0	I1: [0.10, 1.22] I2: [−0.16, 1.01] C: [−0.54, 0.54]
**Mozafaripour et al., 2022** [74]	Knee valgus angle	20	2	1.42 (d)	[0.20, 1.22]	I: −1.31 C: 0.18	I: [−2.00, 0.62] C: [−0.51, 0.87]
**Olson et al., 2011** [77]	Knee frontal plane projection angle	18	1	N/A	N/A	I: −1.13	I: [−1.88, 0.38]
	Knee abduction angle	18	1	N/A	N/A	C: −0.29	I: [−0.95, 0.37]
**Palmer et al., 2015** [79]	Knee valgus angle (single leg squat)	29	2	−0.22 (d)	[−0.50, 0.28]	I1: −0.25 I2: −0.58	I1: [−0.82, 0.31] I2: [−1.17, 0.00]
	Knee valgus angle (single leg landing)	29	2	−0.06 (d)	[−0.43, 0.36]	I1: −0.08 I2: −0.14	I1: [−0.64, 0.48] I2: [−0.73, 0.44]
**Hip and ankle muscle strengthening**							
**Bell et al., 2013** [54]	Medial Knee Displacement	32	2	−1.19 (d)	[−0.95, −0.23]	I: −1.45 C: −0.20	I: [−2.00, −0.91] C: [−0.75, 0.34]
	Knee Valgus	32	2	0.88 (d)	[0.08, 0.80]	I: −0.61 C: 0.26	I: [−1.16, −0.07] C: [−0.29, 0.80]
**Limited ankle RoM**							
**Lower extremity strengthening and ankle mobility training**							
**Howe et al., 2022** [68]	Knee frontal plane projection angle	20	2	0.14 (d)	[−0.43, 0.57]	I1: 0.25 I2: 0.21	I1: [−0.41, 0.91] I2: [−0.51, 0.94]
**Pronated feet**							
**Hip muscle strengthening**							
**Goo et al., 2016** [64]	Navicular drop	18	2	−1.35 (d)	[−1.22, −0.13]	I1: −3.9 I2: −1.89	I1: [−4.22, −2.77] I2: [−2.62, −1.17]
**Foot extrinsic muscle strengthening**							
**Alam et al., 2019** [52]	Dominant limb Navicular drop	28	2	−1.90 (d)	[−1.34, −0.56]	I1: −2.78 I2: −1.02	I1: [−3.36, −2.19] I2: [−1.60, −0.44]
	Non-dominant limb Navicular drop	28	2	−2.10 (d)	[−1.44, −0.66]	I1: −3.21 I2: −0.93	I1: [−1.60, −0.44] I2: [−1.51, −0.35]
**Core and foot muscle strengthening**							
**Sanchez- Rodriguez et al., 2020** [80]	Foot pronation	36	2	−1.06 (d)	[−0.88, −0.18]	I: −0.88 C: 0.00	I: [−1.39, −0.37] C: [−0.51, 0.51]
**Foot intrinsic muscle strengthening**							
**Pabon-Carrasco et al., 2020** [78]	Navicular drop (right foot)	85	2	−0.22 (d)	[−0.32, 0.11]	I: −0.56 C: −0.16	I: [−2.56, −1.88] C: [−0.49, 0.18]
	Navicular drop (left foot)	85	2	−0.71 (d)	[−0.57, −0.14]	I: −0.71 C: −0.91	I: [−1.05, −0.38] C: [−1.25, −0.58]
	Foot pronation (right foot)	85	2	−0.92 (d)	[−0.68, −0.25]	I: −1.02 C: −0.92	I: [−2.58, −1.90] C: [−2.68, −2.02]
	Foot pronation (left foot)	85	2	−1.55 (d)	[−0.99, −0.56]	I: −1.12 C: −2.97	I: [−3.41, −2.74] C: [−3.30, −2.64]
**Flat feet**							
**Foot intrinsic muscle strengthening**							
**Okamura et al., 2020** [76]	Foot pronation	20	2	0.15 (d)	[−0.43, 0.58]	I: −0.68 C: −0.64	I: [−1.37, 0.01] C: [−1.33, 0.04]
	Navicular drop	20	2	−0.28 (d)	[−0.64, 0.37]	I: −0.64 C: −0.39	I: [−1.33, 0.05] C: [−1.08, 0.30]
**Unver et al., 2020** [85]	Navicular drop (right foot)	41	2	−0.93 (d)	[−0.80, −0.13]	I: −0.99 C: −0.06	I: [−3.58, 1.60] C: [−2.69, 2.56]
	Navicular drop (left foot)	41	2	−1.13 (d)	[−0.89, −0.23]	I: −1.12 C: 0.03	I: [−3.48, 1.24] C: [−2.45, 2.50]
	Foot pronation (right foot)	41	2	−0.88 (d)	[−0.77, −0.11]	I: −0.88 C: 0.05	I: [−1.72, −0.04] C: [−1.20, 1.30]
	Foot pronation (left foot)	41	2	−0.95 (d)	[−0.81, −0.14]	I: −0.91 C: 0.09	I: [−1.80, −0.02] C: [−1.18, 1.36]
**Foot extrinsic muscle and lower extremity strengthening**							
**Utsahachant et al., 2023** [86]	Navicular drop	45	3	0.66	[0.16, 0.92]	I1: −1.15 I2: −1.58 C: 0.06	I1: [−1.78, −0.52] I2: [−2.21, −0.95] C: [−0.57, 0.69]
**Brijwasi et al., 2023** [55]	Navicular drop height	49	2	−1.51 (d)	[−1.04, −0.47]	I: −1.96 C: 0.39	I: [−2.07, −1.86] C: [−0.50, −0.28]
	Medial longitudinal arch angle	49	2	1.26	[0.59, 1.17]	I: 2.91 C: 0.40	I: [0.22, 5.61] C: [−3.84, 4.63]

SD: standard deviation; CI: confidence interval; ES: effect size; N/A: not applicable; I: intervention group; I1, I2, I3: intervention groups 1, 2, and 3, respectively; C: control group; d: Cohen’s d effect size; d: 0.2 ≤ d < 0.5: small effect size; 0.5 ≤ d < 0.8: medium effect size; d ≥ 0.8: large effect size; f: Cohen’s f effect size; 0.1 ≤ f < 0.25: small effect size, 0.25 ≤ f < 0.4: medium effect size; f ≥ 0.4: large effect size.

## Supplementary Materials

The following supporting information can be downloaded at https://www.mdpi.com/article/10.3390/sports13020052/s1. Supplementary Table S1: Group Means, Standard Deviations (SD), and *P* Values (*p*) of Studies in Individuals Without a Biomechanical Misalignment; Supplementary Table S2: Group Means, Standard Deviations (SD), and *P* Values (*p*) of Studies in Individuals With a Biomechanical Misalignment.

## Appendix A

**Table A1:** PICO model.

**P**opulation	Healthy adults, aged 18–45 years old
**I**ntervention	Exercise, training programs
**C**omparison	No treatment or sham treatment or other treatment
**O**utcome	Knee valgus, knee abduction, foot pronation or ankle eversion

**Table A2:** Search strategy.

Pubmed:	(((training) OR (exercise) OR (conservative intervention)**)** AND ((foot pronation) OR (foot posture) OR (ankle eversion) OR (navicular drop) OR (knee valgus) OR (knee posture) OR (knee placement) OR (knee abduction) OR (knee frontal projection angle) OR (lower extremity biomechanics) OR (lower extremity kinematics)) NOT ((injury) OR (pain) OR (syndrome) OR (patient) OR (older) OR (elderly) OR (children)))
Web of Science:	(ALL = ((((training) OR (exercise) OR (conservative intervention)) AND ((foot pronation) OR (foot posture) OR (ankle eversion) OR (navicular drop) OR (knee valgus) OR (knee posture) OR (knee placement) OR (knee abduction) OR (knee frontal projection angle) OR (lower extremity biomechanics) OR (lower extremity kinematics)) NOT ((injury) OR (pain) OR (syndrome) OR (patient) OR (older) OR (elderly) OR (children)))
PEDro:	Therapy: fitness training OR strength training OR stretching, Body part: lower leg or knee OR foot or ankle OR thigh or hip, Subdiscipline: sports OR orthopedics OR musculoskeletal, Method: Clinical Trial, Published Since: 2008

## References

[1] Ilahi O.A., Kohl H.W. (1998). Lower extremity morphology and alignment and risk of overuse injury. Clin. J. Sport Med..

[2] Moen M.H., Bongers T., Bakker E.W., Zimmermann W.O., Weir A., Tol J.L., Backx F.J.G. (2012). Risk factors and prognostic indicators for medial tibial stress syndrome. Scand. J. Med. Sci. Sports.

[3] Powers C.M., Bolgla L.A., Callaghan M.J., Collins N., Sheehan F.T. (2012). Patellofemoral pain: Proximal, distal, and local factors, 2nd International Research Retreat. J. Orthop. Sports Phys. Ther..

[4] Abu-Faraj Z.O., Harris G.F., Smith P.A., Hassani S. (2015). Human gait and Clinical Movement Analysis. Wiley Encyclopedia of Electrical and Electronics Engineering.

[5] Hewett T.E., Myer G.D., Ford K.R., Heidt R.S., Colosimo A.J., McLean S.G., Van Den Bogert A.J., Paterno M.V., Succop P. (2005). Biomechanical measures of neuromuscular control and valgus loading of the knee predict anterior cruciate ligament injury risk in female athletes: A prospective study. Am. J. Sports Med..

[6] Oldfather T., Zabala M., Goodlett M., Murrah W. (2020). Knee Valgus Versus Knee Abduction Angle: Comparative Analysis of Medial Knee Collapse Definitions in Female Athletes. J. Biomech. Eng..

[7] Englander Z.A., Cutcliffe H.C., Utturkar G.M., Garrett W.E., Spritzer C.E., DeFrate L.E. (2019). A Comparison of Knee Abduction Angles Measured by a 3D Anatomic Coordinate System Versus Videographic Analysis: Implications for Anterior Cruciate Ligament Injury. Orthop. J. Sports Med..

[8] Mcclay I., Manall K. (1998). A comparison of three-dimensional lower extremity kinematics during running between excessive pronators and normals Introduction and ankle, along with transferring abnormal stresses. Clin. Biomech..

[9] Joseph M., Tiberio D., Baird J.L., Trojian T.H., Anderson J.M., Kraemer W.J., Maresh C.M. (2008). Knee valgus during drop jumps in National Collegiate Athletic Association Division I female athletes: The effect of a medial post. Am. J. Sports Med..

[10] Van Gheluwe B., Kirby K.A., Hagman F. (2005). Effects of Simulated Genu Valgum and Genu Varum on Ground Reaction Forces and Subtalar Joint Function During Gait. J. Am. Podiatr. Med. Assoc..

[11] Hughes G. (2014). A review of recent perspectives on biomechanical risk factors associated with anterior cruciate ligament injury. Res. Sports Med..

[12] Rodrigues P., Chang R., TenBroek T., Van Emmerik R., Hamill J. (2015). Evaluating the coupling between foot pronation and tibial internal rotation continuously using vector coding. J. Appl. Biomech..

[13] Pohl M.B., Messenger N., Buckley J.G. (2006). Changes in foot and lower limb coupling due to systematic variations in step width. Clin. Biomech..

[14] Ghanem I., Massaad A., Assi A., Rizkallah M., Bizdikian A.J., El Abiad R., Seringe R., Mosca V., Wicart P. (2019). Understanding the foot’s functional anatomy in physiological and pathological conditions: The calcaneopedal unit concept. J. Child. Orthop..

[15] Nester C.J., Van Der Linden M.L., Bowker P. (2003). Effect of foot orthoses on the kinematics and kinetics of normal walking gait. Gait Posture.

[16] McDonald S.W., Tavener G. (1999). Pronation and supination of the foot: Confused terminology. Foot.

[17] Souza T.R., Pinto R.Z., Trede R.G., Kirkwood R.N., Fonseca S.T. (2010). Temporal couplings between rearfoot-shank complex and hip joint during walking. Clin. Biomech..

[18] Pinto R.Z.A., Souza T.R., Trede R.G., Kirkwood R.N., Figueiredo E.M., Fonseca S.T. (2008). Bilateral and unilateral increases in calcaneal eversion affect pelvic alignment in standing position. Man. Ther..

[19] Khamis S., Dar G., Peretz C., Yizhar Z. (2015). The Relationship between Foot and Pelvic Alignment while Standing. J. Hum. Kinet..

[20] Menz H.B., Dufour A.B., Riskowski J.L., Hillstrom H.J., Hannan M.T. (2013). Foot posture, foot function and low back pain: The Framingham Foot Study. Rheumatology.

[21] Nascimento D.P., Gonzalez G.Z., Araujo A.C., Costa L.O.P. (2019). Description of low back pain clinical trials in physical therapy: A cross sectional study. Braz. J. Phys. Ther..

[22] Harradine P., Bevan L., Carter N. (2006). An overview of podiatric biomechanics theory and its relation to selected gait dysfunction. Physiotherapy.

[23] Barwick A., Smith J., Chuter V. (2012). The relationship between foot motion and lumbopelvic-hip function: A review of the literature. Foot.

[24] Lee I., Jeon H.G., Ha S., Jeong H., Lee S.Y. (2024). How Medial Tibial Stress Syndrome Is Affected by Alignment, Range of Motion, Strength, and Gait Biomechanics: A Systematic Review and Meta-Analysis. J. Sport Rehabil..

[25] Levinger P., Menz H.B., Morrow A.D., Feller J.A., Bartlett J.R., Bergman N.R. (2012). Foot kinematics in people with medial compartment knee osteoarthritis. Rheumatology.

[26] Kakavas G., Malliaropoulos N.G., Forelli F., Mazeas J., Skarpas G., Maffuli N. (2023). How Subtalar Kinematics Affects Knee Laxity in Soccer Players After Anterior Cruciate Ligament Injury?. Cureus.

[27] Shultz S.J., Nguyen A.D., Levine B.J. (2009). The relationship between lower extremity alignment characteristics and anterior knee joint laxity. Sports Health.

[28] Menz H.B., Dufour A.B., Riskowski J.L., Hillstrom H.J., Hannan M.T. (2013). Association of planus foot posture and pronated foot function with foot pain: The Framingham foot study. Arthritis Care Res..

[29] Kisacik P., Tunay V.B., Bek N., Atay Ö.A., Selfe J., Karaduman A.A. (2021). Short foot exercises have additional effects on knee pain, foot biomechanics, and lower extremity muscle strength in patients with patellofemoral pain. J. Back. Musculoskelet. Rehabil..

[30] Kim T.-H., Lee C.-W., Kim S.-G., An B.-W. (2015). The effect of a pelvis-concentrated exercise program on male college students’ body alignment and foot base pressure. J. Phys. Ther. Sci..

[31] Matthews M., Rathleff M.S., Claus A., McPoil T., Nee R., Crossley K.M., Kasza J., Vicenzino B.T. (2020). Does foot mobility affect the outcome in the management of patellofemoral pain with foot orthoses versus hip exercises? A randomised clinical trial. Br. J. Sports Med..

[32] dos Anjos Rabelo N.D., Costa L.O.P., de Lima B.M., dos Reis A.C., Bley A.S., Fukuda T.Y., Lucareli P.R.G. (2017). Adding motor control training to muscle strengthening did not substantially improve the effects on clinical or kinematic outcomes in women with patellofemoral pain: A randomised controlled trial. Gait Posture.

[33] Ardakani M.K., Wikstrom E.A., Minoonejad H., Rajabi R., Sharifnezhad A. (2019). Hop-stabilization training and landing biomechanics in athletes with chronic ankle instability: A randomized controlled trial. J. Athl. Train..

[34] Huang P.Y., Chen W.L., Lin C.F., Lee H.J. (2014). Lower extremity biomechanics in athletes with ankle instability after a 6-week integrated training program. J. Athl. Train..

[35] McKeon P.O., Paolini G., Ingersoll C.D., Kerrigan D.C., Saliba E.N., Bennett B.C., Hertel J. (2009). Effects of balance training on gait parameters in patients with chronic ankle instability: A randomized controlled trial. Clin. Rehabil..

[36] Jiang L., Zhang L., Huang W., Zeng Q., Huang G. (2022). The effect of proprioception training on knee kinematics after anterior cruciate ligament reconstruction: A randomized control trial. J. Back. Musculoskelet. Rehabil..

[37] Saki F., Shafiee H., Tahayori B., Ramezani F. (2023). The effects of core stabilization exercises on the neuromuscular function of athletes with ACL reconstruction. Sci. Rep..

[38] Milandri G., Sivarasu S. (2021). A Randomized Controlled Trial of Eccentric Versus Concentric Cycling for Anterior Cruciate Ligament Reconstruction Rehabilitation. Am. J. Sports Med..

[39] Sahabuddin F.N.A., Jamaludin N.I., Amir N.H., Shaharudin S. (2021). The effects of hip- And ankle-focused exercise intervention on dynamic knee valgus: A systematic review. PeerJ.

[40] Raghava Neelapala Y.V., Bhagat M., Shah P. (2020). Hip Muscle Strengthening for Knee Osteoarthritis: A Systematic Review of Literature. J. Geriatr. Phys. Ther..

[41] Mozafaripour E., Bayattork M., Shahrbanian S. (2023). Can hip muscle strengthening interventions improve lower extremity kinematics among healthy subjects? A systematic review of randomized controlled trials. Sport Sci. Health.

[42] Cheng J., Han D., Qu J., Liu Z., Huang Y. (2024). Effects of short foot training on foot posture in patients with flatfeet: A systematic review and meta-analysis. J. Back. Musculoskelet. Rehabil..

[43] Oliveira P.M.P., Monteiro J.C.M., Carvalho L.M., de Carvalho F.O. (2024). Strengthening the Intrinsic Muscles of the Foot and Its Action on Foot Posture and Self-Reported Function in Individuals With Lower Limb Injuries: Systematic Review and Meta-Analysis. J. Manip. Physiol. Ther..

[44] ter Stege M.H.P., Dallinga J.M., Benjaminse A., Lemmink K.A.P.M. (2014). Effect of Interventions on Potential, Modifiable Risk Factors for Knee Injury in Team Ball Sports: A Systematic Review. Sports Med..

[45] Wilczyński B., Zorena K., Ślęzak D. (2020). Dynamic knee valgus in single-leg movement tasks. Potentially modifiable factors and exercise training options. A literature review. Int. J. Environ. Res. Public Health.

[46] Page M.J., McKenzie J.E., Bossuyt P.M., Boutron I., Hoffmann T.C., Mulrow C.D., Shamseer L., Tetzlaff J.M., Akl E.A., Brennan S.E. (2021). The PRISMA 2020 statement: An updated guideline for reporting systematic reviews. BMJ.

[47] Cumpston M., Li T., Page M., Chandler J., Welch V., Higgins J.P., Thomas J. (2024). Cochrane Handbook for Systematic Reviews of Interventions, Version 6.5.

[48] Richardson W.S., Wilson M.C., Nishikawa J., Hayward R.S. (1995). The well-built clinical question: A key to evidence-based decisions. ACP J. Club.

[49] The EndNote Team (2013). EndNote [Computer Software], Version X9.

[50] (2023). Sciwheel.

[51] Higgins J.P.T., Savović J., Page M.J., Elbers R.G., Sterne J.A.C., Higgins J.P.T., Thomas J., Chandler J., Cumpston M., Li T., Page M.J., Welch V.A. (2022). Assessing risk of bias in a randomized trial. Cochrane Handbook for Systematic Reviews of Interventions, Version 6.3 (Updated February 2022).

[52] Alam F., Raza S., Moiz J.A., Bhati P., Anwer S., Alghadir A. (2019). Effects of selective strengthening of tibialis posterior and stretching of iliopsoas on navicular drop, dynamic balance, and lower limb muscle activity in pronated feet: A randomized clinical trial. Physician Sportsmed..

[53] Araújo V.L., Souza T.R., do Carmo Carvalhais V.O., Cruz A.C., Fonseca S.T. (2017). Effects of hip and trunk muscle strengthening on hip function and lower limb kinematics during step-down task. Clin. Biomech..

[54] Bell D.R., Oates D.C., Clark M.A., Padua D.A. (2013). Two- and 3-dimensional knee valgus are reduced after an exercise intervention in young adults with demonstrable valgus during squatting. J. Athl. Train..

[55] Brijwasi T., Borkar P. (2023). A comprehensive exercise program improves foot alignment in people with flexible flat foot: A randomised trial. J. Physiother..

[56] Chappell J.D., Limpisvasti O. (2008). Effect of a neuromuscular training program on the kinetics and kinematics of jumping tasks. Am. J. Sports Med..

[57] da Silva Neto W.C., Lopes A.D., Ribeiro A.P. (2022). Gait Retraining With Visual Biofeedback Reduces Rearfoot Pressure and Foot Pronation in Recreational Runners. J. Sport Rehabil..

[58] Dawson S.J., Herrington L. (2015). Improving single-legged-squat performance: Comparing 2 training methods with potential implications for injury prevention. J. Athl. Train..

[59] de Marche Baldon R., Lobato D.F.M., Yoshimatsu A.P., dos Santos A.F., Francisco A.L., Santiago P.R.P., Serrão F.V. (2013). Effect of Plyometric Training on Lower Limb Biomechanics in Females. Clin. J. Sport Med..

[60] De Marche Baldon R., Serrão F.V., Silva R.S., Piva S.R. (2014). Effects of functional stabilization training on pain, function, and lower extremity biomechanics in women with patellofemoral pain: A randomized clinical trial. J. Orthop. Sports Phys. Ther..

[61] Dunn M.D., Claxton D.B., Fletcher G., Wheat J.S., Binney D.M. (2018). Effects of running retraining on biomechanical factors associated with lower limb injury. Hum. Mov. Sci..

[62] Ericksen H.M., Thomas A.C., Gribble P.A., Armstrong C., Rice M., Pietrosimone B. (2016). Jump-landing biomechanics following a 4-week real-time feedback intervention and retention. Clin. Biomech..

[63] Ghanati H.A., Letafatkar A., Shojaedin S., Hadadnezhad M., Schöllhorn W.I. (2022). Comparing the Effects of Differential Learning, Self-Controlled Feedback, and External Focus of Attention Training on Biomechanical Risk Factors of Anterior Cruciate Ligament (ACL) in Athletes: A Randomized Controlled Trial. Int. J. Environ. Res. Public Health.

[64] Goo Y.-M., Kim T.-H., Lim J.-Y. (2016). The effects of gluteus maximus and abductor hallucis strengthening exercises for four weeks on navicular drop and lower extremity muscle activity during gait with flatfoot. J. Phys. Ther. Sci..

[65] Herman D.C., Oñate J.A., Weinhold P.S., Guskiewicz K.M., Garrett W.E., Yu B., Padua D.A. (2009). The effects of feedback with and without strength training on lower extremity biomechanics. Am. J. Sports Med..

[66] Herman D.C., Weinhold P.S., Guskiewicz K.M., Garrett W.E., Yu B., Padua D.A. (2008). The effects of strength training on the lower extremity biomechanics of female recreational athletes during a stop-jump task. Am. J. Sports Med..

[67] Herrington L. (2010). The effects of 4 weeks of jump training on landing knee valgus and crossover hop performance in female basketball players. J. Strength Cond. Res..

[68] Howe L.P., Bampouras T.M., North J.S., Waldron M. (2022). Improved ankle mobility following a 4-week training program affects landing mechanics: A randomized controlled trial 2. J. Strength Cond. Res..

[69] Jeong J., Choi D.H., Song Y., Shin C.S. (2020). Muscle Strength Training Alters Muscle Activation of the Lower Extremity during Side-Step Cutting in Females. J. Mot. Behav..

[70] Jeong J., Choi D.H., Shin C.S. (2021). Core Strength Training Can Alter Neuromuscular and Biomechanical Risk Factors for Anterior Cruciate Ligament Injury. Am. J. Sports Med..

[71] Kato S., Urabe Y., Kawamura K. (2008). Alignment control exercise changes lower extremity movement during stop movements in female basketball players. Knee.

[72] Lynn S.K., Padilla R.A., Tsang K.K.W. (2012). Differences in Static-and Dynamic-Balance Task Performance After 4 Weeks of Intrinsic-Foot-Muscle Training: The Short-Foot Exercise Versus the Towel-Curl Exercise. J. Sport Rehabil..

[73] McCurdy K., Walker J., Saxe J., Woods J. (2012). The effect of short-term resistance training on hip and knee kinematics during vertical drop jumps. J. Strength. Cond. Res..

[74] Mozafaripour E., Seidi F., Minoonejad H., Bayattork M., Khoshroo F. (2022). The effectiveness of the comprehensive corrective exercise program on kinematics and strength of lower extremities in males with dynamic knee valgus: A parallel-group randomized wait-list controlled trial. BMC Musculoskelet. Disord..

[75] Mulligan E.P., Cook P.G. (2013). Effect of plantar intrinsic muscle training on medial longitudinal arch morphology and dynamic function. Man. Ther..

[76] Okamura K., Fukuda K., Oki S., Ono T., Tanaka S., Kanai S. (2020). Effects of plantar intrinsic foot muscle strengthening exercise on static and dynamic foot kinematics: A pilot randomized controlled single-blind trial in individuals with pes planus. Gait Posture.

[77] Olson T.J., Chebny C., Willson J.D., Kernozek T.W., Straker J.S. (2011). Comparison of 2D and 3D kinematic changes during a single leg step down following neuromuscular training. Phys. Ther. Sport.

[78] Pabón-Carrasco M., Castro-Méndez A., Vilar-Palomo S., Jiménez-Cebrián A.M., García-Paya I., Palomo-Toucedo I.C. (2020). Randomized clinical trial: The effect of exercise of the intrinsic muscle on foot pronation. Int. J. Environ. Res. Public Health.

[79] Palmer K., Hebron C., Williams J.M. (2015). A randomised trial into the effect of an isolated hip abductor strengthening programme and a functional motor control programme on knee kinematics and hip muscle strength Rehabilitation, physical therapy and occupational health. BMC Musculoskelet. Disord..

[80] Sánchez-Rodríguez R., Valle-Estévez S., Fraile-García P.A., Martínez-Nova A., Gómez-Martín B., Escamilla-Martínez E. (2020). Modification of pronated foot posture after a program of therapeutic exercises. Int. J. Environ. Res. Public Health.

[81] Sasaki S., Tsuda E., Yamamoto Y., Maeda S., Kimura Y., Fujita Y., Ishibashi Y. (2019). Core-muscle training and neuromuscular control of the lower limb and trunk. J. Athl. Train..

[82] Snyder K.R., Earl J.E., O’Connor K.M., Ebersole K.T. (2009). Resistance training is accompanied by increases in hip strength and changes in lower extremity biomechanics during running. Clin. Biomech..

[83] Sulowska I., Oleksy Ł., Mika A., Bylina D., Sołtan J. (2016). The influence of plantar short foot muscle exercises on foot posture and fundamental movement patterns in long-distance runners, a non-randomized, non-blinded clinical trial. PLoS ONE.

[84] Tate J.J., Milner C.E., Fairbrother J.T., Zhang S. (2013). The effects of a home-based instructional program aimed at improving frontal plane knee biomechanics during a jump-landing task. J. Orthop. Sports Phys. Ther..

[85] Unver B., Erdem E.U., Akbas E. (2020). Effects of short-foot exercises on foot posture, pain, disability, and plantar pressure in pes planus. J. Sport Rehabil..

[86] Utsahachant N., Sakulsriprasert P., Sinsurin K., Jensen M.P., Sungkue S. (2023). Effects of short foot exercise combined with lower extremity training on dynamic foot function in individuals with flexible flatfoot: A randomized controlled trial. Gait Posture.

[87] Ford K., Nguyen A.-D., Dischiavi S., Hegedus E., Zuk E., Taylor J. (2015). An evidence-based review of hip-focused neuromuscular exercise interventions to address dynamic lower extremity valgus. Open Access J. Sports Med..

[88] Padua D., Beutler A., DeMaio M. (2007). Anterior tibial shear force and knee valgus angle are influenced by lower extremity kinematics, muscle strength, and landing technique. J. Orthop. Sports Phys. Ther..

[89] Arendt E.A. (2007). Core strengthening. Instr. Course Lect..

[90] De Blaiser C., Roosen P., Willems T., Danneels L., Bossche L.V., De Ridder R. (2018). Is core stability a risk factor for lower extremity injuries in an athletic population? A systematic review. Phys. Ther. Sport.

[91] Chuter V.H., de Jonge X.A.J. (2012). Proximal and distal contributions to lower extremity injury: A review of the literature. Gait Posture.

[92] Willson J.D., Ireland M.L., Davis I. (2006). Core strenght and lower extremity alignment during single leg squats. Med. Sci. Sports Exerc..

[93] Ireland M.L., Bolgla L.A., Noehren B., Bolgla L.A., Noehren B. (2018). Gender Differences in Core Strength and Lower Extremity Function During Static and Dynamic Single-Leg Squat Tests. ACL Injuries in the Female Athlete.

[94] Newsham K.R. (2010). Strengthening the Intrinsic Foot Muscles. Athl. Ther. Today.

[95] Reiner S.L. (2021). Attentional Focus Cueing: How and When to Use Internal and External Focus Cues to Optimize Exercise Performance. ACSM’s Health Fit. J..

[96] Becker K.A., Fairbrother J.T., Couvillion K.F. (2020). The effects of attentional focus in the preparation and execution of a standing long jump. Psychol. Res..

[97] Agresta C., Brown A. (2015). Gait retraining for injured and healthy runners using augmented feedback: A systematic literature review. J. Orthop. Sports Phys. Ther..

[98] Richards R., van den Noort J.C., Dekker J., Harlaar J. (2017). Gait Retraining With Real-Time Biofeedback to Reduce Knee Adduction Moment: Systematic Review of Effects and Methods Used. Arch. Phys. Med. Rehabil..

[99] Rynne R., Le Tong G., Cheung R.T.H., Constantinou M. (2022). Effectiveness of gait retraining interventions in individuals with hip or knee osteoarthritis: A systematic review and meta-analysis. Gait Posture.

[100] Hara S., Kitano M., Kudo S. (2023). The effects of short foot exercises to treat flat foot deformity: A systematic review. J. Back. Musculoskelet. Rehabil..

[101] Medina Mckeon J.M., Hertel J. (2009). Sex Differences and Representative Values for 6 Lower Extremity Alignment Measures. J. Athl. Train..

