# Supplementary material for: Exercise Modalities for Improving Frontal Plane Knee and Foot Posture in Healthy Adults: A Systematic Review
Source: Sports (Basel). 2025 Feb 11;13(2):52. doi: 10.3390/sports13020052 (PMC11861904; doi:10.3390/sports13020052)
Supplement: Supplementary file 1 [file sports-13-00052-s001.zip › Supplementary Table S1.pdf]

Supplementary Table S1. Group Means, Standard Deviations (SD), and *P* Values (*p*) of Studies in Individuals Without a Biomechanical Misalignment

Author's Name	Parameter	Sample Size	Group 1 Mean, SD	Group 2 Mean, SD	Group 3 Mean, SD	Group 4 Mean, SD	*P* value Within Group	*P* value Between Groups
**Hip and Lower Extremity Muscles Strengthening Training**								
**Snyder et al., 2009** [82]	Knee Abduction Angle	13	Pre: Mean= 3.5, SD= 2.1 Post: Mean= 3.9, SD= 2.1 (I)	-	-	-	I: N/S	N/A
	Foot Eversion Angle	13	Pre: Mean= 9.8, SD= 3.3 Post: Mean= 8.1, SD= 3.9 (I)	-	-	-	I: 0.05*	N/A
**Herman et al., 2008** [66]	Knee Valgus Angle	66	Pre: Mean= -0.33, SD= 3.8 Post: Mean= -0,27, SD= 3.8 (I)	Pre: Mean= 0.29, SD= 4.53 Post: Mean= 0.68, SD= 3.35 (C)	-	-	I: N/S C: N/S	N/S
**McCurdy et al., 2012** [73]	Knee Valgus Angle (Unilateral Drop Jump)	20	Pre: Mean= 10.8, SD= 1.5 Post: Mean= 10.1, SD= 1.9 (I)	Pre: Mean= 11.3, SD= 2.1 Post: Mean= 12.3, SD= 2.2 (C)	-	-	I: N/S C: N/S	N/S
	Knee Valgus Angle (Bilateral Drop Jump)	20	Pre: Mean= 12.0, SD= 2.3 Post: Mean= 13.0,	Pre: Mean= 12.2, SD= 2.0 Post: Mean= 12.9,	-	-	I: N/S C: N/S	N/S

			SD= 2.5 (I)	SD= 2.4 (C)				
**Araujo et al., 2017** [53]	Knee Abduction Angle	34	Pre: Mean= -1.5, SD= 7.01 Post: Mean=-1.06, SD= 4.02 (I)	Pre: Mean= -0.65, SD= 5.72 Post: Mean= 1.85, SD= 2.88 (C)	-	-	I: N/S C: N/S	N/S
	Foot Eversion	34	Pre: Mean= -11.29, SD= 4.53 Post: Mean=-10.97, SD= 5.34 (I)	Pre: Mean= -13.16, SD= 5.45 Post: Mean=-14.74, SD= 6.40 (C)	-	-	I: N/S C: N/S	N/S
**Jeong et al., 2020** [69]	Knee Valgus Angle	25	Pre: Mean= 1.2, SD= 2.1 Post: Mean= 1.7, SD= 3.3 (I)	Pre: Mean= 0.8, SD= 3.0 Post: Mean= 1.5, SD= 2.4 (C)	-	-	I: N/S C: N/S	N/S
**Core Muscles Strengthening**								
**Jeong et al., 2021** [70]	Knee Valgus Angle	48	Pre: Mean= 1.3, SD= 1.6 Post: Mean= 0.7 , SD= 1.5 (I)	Pre: Mean= 1.0, SD= 4.1 Post: Mean= 1.6, SD= 2.5 (C)	-	-	I: 0.03* C: N/S	<0.05*
**Sasaki et al., 2019** [81]	Knee Valgus Angle (Jump Landing)	17	Pre: Mean= 5.8, SD= 2.0 Post: Mean= 2.6, SD= 2.89 (I)	Pre: Mean= 5.3, SD= 1.41 Post: Mean= 6.6, SD= 4.15(C)	-	-	I: N/S C: N/S	N/A

	Knee Valgus Angle (Single Leg Squat)	17	Pre: Mean= 5.0, SD= 3.04 Post: Mean= 2.2, SD= 1.70 (I)	Pre: Mean= 2.9, SD=1.78 Post: Mean= 3.4, SD= 2.67 (C)	-	-	I: 0.008** C: N/S	N/A
**Foot Muscles Strengthening**								
**Mulligan et al., 2013** [75]	Navicular Drop	21	Pre: Mean= 12.7, SD= 6.0 Post: Mean= 10.9, SD= 5.5 (I)		-	-	I: 0.01*	N/A
**Lynn et al., 2012** [72]	Navicular Height	24	Pre: Mean= 44.7, SD= 3.2 Post: Mean= 42.9, SD= 4.6 (I1)	Pre: Mean= 39.5, SD= 8.3 Post: Mean= 39.5, SD= 5.8 (I2)	Pre: Mean= 41.3, SD= 7.0 Post: Mean= 40.9, SD= 4.3 (C)	-	I1: N/S 12: N/S C: N/S	N/A
**Sulowska et al., 2016** [83]	Foot Pronation	25	Pre: Mean= 3, SD= 2.22 Post: Mean= 2, SD= 1.40 (I1)	Pre: Mean= 3.0, SD= 2.58 Post: Mean= 2-0, SD= 1.55 (I2)	-	-	I1: N/S I2. N/S	N/A
**Technique Training**								
**De Marche Baldon et al., 2013** [59]	Knee abduction angle	36	Pre: Mean= -9.23, SD= 2.83 Post: Mean= -5.78, SD= 2.74 (I)	Pre: Mean= -11.74, SD= 5.83 Post: Mean=-12.0, SD= 8.53 (C)	-	-	I: 0.01* C: N/A	0.04*

**Herrington et al. , 2010** [67]	Knee valgus angle (drop jump task) (right leg)	15	Mdiff= 12.3 (I)	-	-	-	I: 0.002**	N/A
	Knee valgus angle (drop jump task) (left leg)	15	Mdiff= 9.8 (I)	-	-	-	I: 0.001***	N/A
	Knee valgus angle (jump-shot task) (right leg)	15	Mdiff= 4.3 (I)	-	-	-	I: 0.03*	N/A
	Knee valgus angle (jump-shot task) (left leg)	15	Mdiff= 4.5 (I)	-	-	-	I: 0.01**	N/A
**Ghanati et al.**	Knee abduction angle	42	Pre: Mean= 13.51, SD= 2.75 Post: Mean= 12.34, SD= 1.47 (I1)	Pre: Mean= 13.54, SD= 3.07, Post: Mean= 10.22, SD= 2.43 (I2)	Pre: Mean= 13.28 SD= 2.44, Post: Mean= 6.86, SD= 4.38 (I3)	Pre: Mean= 13.0, SD: 3.06, Post: Mean= 13.73, SD= 2.28 (C)	I1: N/S I2: 0.001*** I3:0.001*** C: N/S	I3- C: 0.001*** I3-I1: 0.001*** I2-I1: 0.001***
**Kato et al.**	Knee abduction angle	20	Pre: Mean= 36.9, SD= 19.5 Post: Mean= 23.2, SD= 20.01 (I)	Pre: Mean= 40.1, SD= 23.6 Post: Mean= 36.1, SD= 23.8 (C)	-	-	I: <0.05* C: N/S	< 0.05*
**Dawson et al.**	Knee frontal plane projection angle	17	Pre: Mean= 12.76, SD= 4.44 Post: Mean=	Pre: Mean= 13.34, SD= 4.46 Post:	-	-	I1: 0.001*** I2: 0. 003**	N/S

			6.25, SD= 3.19 (I1)	Mean= 7.12, SD= 2.13 (I2)				
**Herman et al.**	Knee valgus angle	58	Pre: Mean= 0.09, SD= 3.83 Post: Mean= -0,46, SD= 3.71 (I1)	Pre: Mean= -0.63, SD= 3.42 Post: Mean= -0.14, SD= 3.89 (I2)	-	-	N/S	N/S
**Gait/running Retraining**								
**Da Silva Neto et al.**	Foot Posture Index (Right Foot)	24	Pre: Mean= 7.9, SD= 4.3 Post: Mean= 6.6, SD= 4.1 (I)	Pre: Mean= 3.7 SD= 4.2 Post: Mean= 3.7, SD= 4.2 (C)	-	-	I: 0.02* C: N/S	0.02*
	Foot Posture Index (Left Foot)	24	Pre: Mean= 8.2, SD= 3.9 Post: Mean= 6.7, SD= 3.8 (I)	Pre: Mean= 4.7 SD= 3.8 Post: Mean= 4.7, SD= 3.8 (C)	-	-	I: 0.03* C: N/S	0.03*
**Dunn et al.**	Foot eversion angle	20	Pre: Mean= 1.28, SD= 5.81 Post: Mean= 2.26, SD= 5.14 (I)	Pre: Mean= 6.59, SD= 5.24 Post: Mean= 4.16, SD= 4.25 (C)	-	-	I: N/S C: N/S	N/S
**Combined Trainings**								
**De Marche Baldon et al.**	Knee abduction angle	28	Pre: Mean= -6.86, SD= 5.20 Post: Mean= -1.49,	Pre: Mean= -8.20, SD=5.86 Post: Mean= -9,6,	-	-	I: < 0.001*** C: N/S	0.002**

			SD= 3.56 (I)	SD= 6.21 (C)				
**Chappell et al.**	Knee Abduction Angle (Drop Jump)	30	Pre: Mean= 25.7, SD= 14.7 Post: Mean= 24.2, SD= 10.9 (I)	-	-	-	I: N/S	N/A
	Knee Abduction Angle (Stop Jump)	30	Pre: Mean= 28.4, SD= 10.8 Post: Mean= 26.5, SD= 12.1 (I)	-	-	-	I: N/S	N/A

SD: Standard Deviation; *p*-value: Statistical significance level; N/S: Not Significant; N/A: Not Applicable; I: Intervention Group; I1, I2, I3: Intervention Group 1, 2, and 3, respectively; C: Control Group; Mdiff: Mean Difference; *$p < 0.05$: significant difference; **$p < 0.01$: high significant difference; **$p < 0.001$: very high significant difference.
