# Supplementary material for: Exercise Modalities for Improving Frontal Plane Knee and Foot Posture in Healthy Adults: A Systematic Review
Source: Sports (Basel). 2025 Feb 11;13(2):52. doi: 10.3390/sports13020052 (PMC11861904; doi:10.3390/sports13020052)
Supplement: Supplementary file 1 [file sports-13-00052-s001.zip › Supplementary Table S2.pdf]

Supplementary Table S2. Group Means, Standard Deviations (SD), and *P* Values (*p*) of Studies in Individuals With a Biomechanical Misalignment

Author's Name	Parameter	Sample Size	Group 1 Mean, SD	Group 2 Mean, SD	Group 3 Mean, SD	Group 4 Mean,SD	P value Within Group	P value Between groups
				**Valgus knee**				
**Technique trainings**								
**Tate et al., 2013** [84]	Knee abduction angle	26	Pre: Mean= -10.1, SD=4.1 Post: Mean= -10.3, SD=4.8 (I)	Pre: Mean= -7.9, SD=2.7 Post: Mean= -9.3, SD=4.8 (C)	-	-	I: N/A C: N/A	N/S
**Ericksen et al., 2016** [62]	Knee abduction angle	45	Pre: Mean= 1.2, SD=2.7 Post: Mean= -0.4, SD=2.0 (I1)	Pre: Mean= 2.2, SD=4.0 Post: Mean= 0.7, SD=2.7 (I2)	Pre: Mean= 0.7, SD=4.8 Post: Mean= 0.7, SD= 4.8 (C)	-	N/S	N/S
**Mozafaripour et al., 2022** [74]	Knee valgus angle	20	Pre: Mean= -19.71, SD=7.48 Post: Mean= -11.24, SD=3.6 (I)	Pre: Mean= -18.51, SD=7.15 Post: Mean= -19.84, SD=7.42 (C)	-	-	N/A	0.001***
**Olson et al., 2011** [77]	Knee frontal plane projection angle	18	Pre: Mean= -8.0, SD= 3.0 Post: Mean=-3.4, SD=4.9 (I)	-	-	-	I: 0.001***	

	Knee abduction angle	18	Pre: Mean= 1.4, SD= 5.2 Post: Mean=0.0, SD=4.6 (I)	-	-	-	I: N/S	N/A
**Palmer et al., 2015** [79]	Knee valgus angle (single leg squat)	29	Pre: Mean= -19.4, SD=19.8 Post: Mean= -13.8, SD=23.9 (I1)	Pre: Mean= -16.2, SD=10.2 Post: Mean=-6.2, SD=19.8 (I2)	-	-	I1: N/S I2: N/S	N/S
	Knee valgus angle (single leg landing)	29	Pre: Mean= -9.8 SD=11.9 Post: Mean= -8.9, SD=10.6 (I1)	Pre: Mean= -5.2, SD=9.6 Post: Mean= -3.6, SD=12.3 (I2)	-	-	I1: N/S I2: N/S	N/S
**Hip and ankle muscles strengthening**								
**Bell et al., 2013** [54]	Medial Knee Displacement	32	Pre: Mean= 2.2, SD= 3.0 Post: Mean=-1.8, SD=2.4 (I)	Pre: Mean= 2.4, SD=2.5 Post: Mean=1.8, SD=3.3 (C)	-	-	I: 0.02*	N/A
	Knee Valgus	32	Pre: Mean= -7.7, SD= 7.2 Post: Mean=-2.7, SD=8.9 (I)	Pre: Mean= -0.7, SD= 6.8 Post: Mean=-2.6, SD=7.9 (C)	-	-	I: 0.001***	N/A
**Limited Ankle Rom**								
**Lower extremity strengthening and ankle mobility training**								

**Howe et al., 2022** [68]	Knee frontal plane projection angle	20	Pre: Mean= 199.3, SD=22.7 Post: Mean= 204.9, SD=22.3 (I1)	Pre: Mean= 195.5, SD=13.2 Post: Mean= 198.4, SD=14.1 (I2)	-	-	N/S	N/S
				**Pronated feet**				
**Hip muscles strengthening**								
**Goo et al., 2016** [64]	Navicular drop	18	Pre: Mean= 11.9, SD= 2.0 Post: Mean= 5.5, SD=1.6 (I1)	Pre: Mean= 11.4, SD= 1.4 Post: Mean= 7.6, SD=2.3 (I2)	-	-	I1: <0.05* I2: N/S	N/S
**Foot extrinsic muscles strengthening**								
**Alam et al., 2019** [52]	Dominant Limb Navicular Drop	28	Pre: Mean= 13.5, SD=2.02 Post: Mean=8.5, SD=1.44 (I1)	Pre: Mean= 13.0, SD= 1.79 Post: Mean=11.3, SD=1.50 (I2)	-	-	I1: < 0.001*** I2: N/S	N/S
	Non-Dominant Limb Navicular Drop	28	Pre: Mean= 14.7, SD= 2.09 Post: Mean=8.7, SD=1.50 (I1)	Pre: Mean= 13.5, SD= 2.13 Post: Mean=11.6, SD=1.93 (I2)	-	-	I1: < 0.001*** I2: N/S	N/S
**Core and foot muscle strengthening**								
**Sanchez-Rodriguez et al., 2020** [80]	Foot pronation	36	Pre: Mean= 8.1, SD=1.7 Post: Mean= 6.4, SD=2.1 (I)	Pre: Mean= 8.0, SD=1.2 Post: Mean= 8.0, SD=1.2 (C)	-	-	I: 0.001*** C: N/S	0.001***
**Foot intrinsic muscles strengthening**								

**Pabon-Carrasco et al., 2020** [78]	Navicular drop (right foot)	85	Pre: Mean= 0.79, SD=0.08 Post: Mean= 0.63, SD=0.06 (I)	Pre: Mean= 0.67, SD=0.06 Post: Mean= 0.59, SD=0.54 (C)	-	-	I: N/A C: N/A	N/S
	Navicular drop (left foot)	85	Pre: Mean= 0.70, SD=0.06 Post: Mean= 0.49, SD=0.32 (I)	Pre: Mean= 0.65, SD=0.07 Post: Mean= 0.59, SD=0.06(C)	-	-	I: N/A C: N/A	N/S
	Foot pronation (FPI-6) (right foot)	85	Pre: Mean= 6.77, SD=0.62 Post: Mean= 5.37, SD=0.63 (I)	Pre: Mean= 6.35 SD=0.31 Post: Mean= 5.43, SD=0.44(C)	-	-	I: N/A C: N/A	N/S
	Foot pronation (FPI-6) (left foot)	85	Pre: Mean= 6.94 SD=0.52 Post: Mean= 5.09 SD=0.66 (I)	Pre: Mean= 6.27, SD=0.22 Post: Mean= 5.19, SD=0.42 (C)	-	-	I: N/A C: N/A	N/S
				**Flat feet**				
**Foot intrinsic muscles strengthening**								
**Okamura et al., 2020** [76]	Foot pronation (FPI-6)	20	Pre: Mean= 9.7, SD=1.9 Post: Mean= 8.5, SD=1.6 (I)	Pre: Mean= 9.0, SD=2.1 Post: Mean= 7.5, SD=2.5 (C)	-	-	I: 0.01** C: 0.02*	N/A
	Navicular drop	20	Pre: Mean= 12.5, SD=3.3	Pre: Mean= 10.9, SD=2.9	-	-	I: N/S C: < 0.05*	N/A

			Post: Mean= 10.6, SD=2.5 (I)	Post: Mean= 9.8, SD=2.7 (C)				
**Unver et al., 2020** [85]	Navicular drop (right foot)	41	Pre: Mean= 16.47, SD=5.45 Post: Mean= 10.85, SD=5.92 (I)	Pre: Mean= 17.25, SD=5.31 Post: Mean= 16.90, SD=5.90(C)	-	-	I: <0.001*** C: N/S	N/A
	Navicular drop (left foot)	41	Pre: Mean= 17.38, SD=5.85 Post: Mean= 11.57, SD=4.41 (I)	Pre: Mean= 16.30, SD=4.97 Post: Mean= 16.45, SD=5.59 (C)	-	-	I: <0.001*** C: N/S	N/A
	Foot pronation (right foot)	41	Pre: Mean= 8.95, SD=1.46 Post: Mean= 7.33, SD=2.15 (I)	Pre: Mean= 8.40, SD=1.95 Post: Mean= 8.50, SD=2.03 (C)	-	-	I: 0.001*** C: N/S	N/A
	Foot pronation (left foot)	41	Pre: Mean= 8.76, SD=1.84 Post: Mean= 7.09, SD=2.44 (I)	Pre: Mean= 8.10, SD=1.61 Post: Mean= 8.25, SD=1.48 (C)	-	-	I: 0.002** C: N/S	N/A
**Foot extrinsic muscles and lower extremity strengthening**								
**Utsahachant et al., 2023** [86]	Navicular drop	45	Pre: Mean= 15.53, SD=2.75 Post: Mean= 11.12, SD=4.38 (I1)	Pre: Mean= 15.47, SD=3.16 Post: Mean= 10.76, SD=2.77 (I2)	Pre: Mean= 14.24, SD=3.60 Post: Mean= 14.45, SD=	-	I1: <0.001*** I2: <0.001***	I1-12: N/S, I1-C: 0.04*, I2-C: 0.02*

					3.47 (C)			
**Brijwasi et al., 2023** [55]	Navicular Drop Height	49	Pre: Mean= 1.5, SD= 0.3 Post: Mean=1.0, SD=0.2 (I)	Pre: Mean= 1.6, SD= 0.2 Post: Mean=1.5, SD=0.3 (C)	-	-	N/A	N/A
	Medial Longitudinal Arch Angle	49	Pre: Mean= 120, SD= 6 Post: Mean=139, SD=7 (I)	Pre: Mean= 115, SD= 11 Post: Mean=119, SD=9 (C)	-	-	N/A	N/A

SD: Standard Deviation; *p*-value: Statistical significance level; N/S: Not Significant; N/A: Not Applicable; I: Intervention Group; I1, I2, I3: Intervention Group 1, 2, and 3, respectively; C: Control Group; Mdiff: Mean Difference; *$p < 0.05$: significant difference; **$p < 0.01$: high significant difference; **$p < 0.001$: very high significant difference.
